# Clinical potential of circulating free DNA and circulating tumour cells in patients with metastatic non‐small‐cell lung cancer treated with pembrolizumab

**DOI:** 10.1002/1878-0261.13094

**Published:** 2021-09-23

**Authors:** Patricia Mondelo‐Macía, Jorge García‐González, Luis León‐Mateos, Urbano Anido, Santiago Aguín, Ihab Abdulkader, María Sánchez‐Ares, Alicia Abalo, Aitor Rodríguez‐Casanova, Ángel Díaz‐Lagares, Ramón Manuel Lago‐Lestón, Laura Muinelo‐Romay, Rafael López‐López, Roberto Díaz‐Peña

**Affiliations:** ^1^ Liquid Biopsy Analysis Unit Translational Medical Oncology (Oncomet) Health Research Institute of Santiago (IDIS) Santiago de Compostela Spain; ^2^ Universidade de Santiago de Compostela (USC) Santiago de Compostela Spain; ^3^ Department of Medical Oncology Complexo Hospitalario Universitario de Santiago de Compostela (SERGAS) Santiago de Compostela Spain; ^4^ Translational Medical Oncology (Oncomet) Health Research Institute of Santiago (IDIS) Santiago de Compostela Spain; ^5^ Centro de Investigación Biomédica en Red de Cáncer (CIBERONC) Madrid Santiago de Compostela Spain; ^6^ Department of Pathology Complexo Hospital Universitario de Santiago de Compostela (SERGAS) Universidade de Santiago de Compostela Santiago de Compostela Spain; ^7^ Cancer Epigenomics Translational Medical Oncology (Oncomet) Health Research Institute of Santiago (IDIS) Santiago de Compostela Spain; ^8^ Roche‐CHUS Joint Unit Translational Medical Oncology (Oncomet) Health Research Institute of Santiago (IDIS) Santiago de Compostela Spain

**Keywords:** biomarkers, cfDNA, CTCs, immunotherapy, NSCLC, PD‐L1

## Abstract

Immune checkpoint inhibitors, such as pembrolizumab, are revolutionizing therapeutic strategies for different cancer types, including non‐small‐cell lung cancer (NSCLC). However, only a subset of patients benefits from this therapy, and new biomarkers are needed to select better candidates. In this study, we explored the value of liquid biopsy analyses, including circulating free DNA (cfDNA) and circulating tumour cells (CTCs), as a prognostic or predictive tool to guide pembrolizumab therapy. For this purpose, a total of 109 blood samples were collected from 50 patients with advanced NSCLC prior to treatment onset and at 6 and 12 weeks after the initiation of pembrolizumab. Plasma cfDNA was measured using *hTERT* quantitative PCR assay. The CTC levels at baseline were also analysed using two enrichment technologies (CellSearch^®^ and Parsortix systems) to evaluate the efficacy of both approaches at detecting the presence of programmed cell death ligand 1 on CTCs. Notably, patients with high baseline *hTERT* cfDNA levels had significantly shorter progression‐free survival (PFS) and overall survival (OS) than those with low baseline levels. Moreover, patients with unfavourable changes in the *hTERT* cfDNA levels from baseline to 12 weeks showed a higher risk of disease progression. Additionally, patients in whom CTCs were detected using the CellSearch^®^ system had significantly shorter PFS and OS than patients who had no CTCs. Finally, multivariate regression analyses confirmed the value of the combination of CTCs and cfDNA levels as an early independent predictor of disease progression, identifying a subgroup of patients who were negative for CTCs, who presented low levels of cfDNA and who particularly benefited from the treatment.

## Graphical abstract

In the present work, we demonstrated that liquid biopsy, through the combinatory analyses of circulating free DNA (cfDNA) and circulating tumour cells and the monitoring of cfDNA during the treatment, could help to select advanced non‐small‐cell lung cancer patients who will benefit from pembrolizumab treatment as first line, providing a promising non‐invasive strategy for improving the clinical management of this tumour.

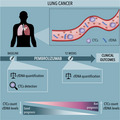

**Abbreviations**
APC: allophycocyanin
AUC: area under the curve
cfDNA: circulating free DNA
CKs: cytokeratins
Cq: quantification values
CR: complete response
CTCs: circulating tumour cells
DAPI: dye 4′,6‐diamidino‐2‐phenylindole
ECOG PS: Eastern Cooperative Oncology Group Performance Status
FDA: Food and Drug Administration
FLU: fluorescein
ICIs: immune checkpoint inhibitors
IHC: immunohistochemistry
NSCLC: non‐small‐cell lung cancer
ORR: objective response rate
OS: overall survival
PD: progressive disease
PD‐1: programmed cell death protein 1
PD‐L1: programmed cell death ligand 1
PE: phycoerythrin
PFS: progression‐free survival
PR: partial response
qPCR: quantitative PCR
ROC: receiver operating characteristic
SD: stable disease
TMB: tumour mutational burden
TPS: tumour proportion score

## 1 Introduction

Lung cancer is the most commonly diagnosed cancer, ranking first in morbidity and mortality rates among malignant tumours worldwide [1]. Under normal physiological conditions, immune checkpoint proteins are crucial for the maintenance of self‐tolerance, protecting tissues from damage when the immune system responds to infections. However, the expression of immune checkpoint proteins is dysregulated by tumours as an immune resistance mechanism [2]. Over the past decade, immunotherapy has become a milestone in the treatment of non‐small‐cell lung cancer (NSCLC), which accounts for 80–90% of all lung cancer cases [3].

Currently, immune checkpoint inhibitors (ICIs) target both programmed cell death protein 1 (PD‐1) and programmed cell death ligand 1 (PD‐L1). Pembrolizumab is a humanized IgG4 monoclonal antibody that inhibits the PD‐1 receptor. When used as a monotherapy, it is the standard first‐line treatment for selected patients with metastatic NSCLC presenting high PD‐L1 tissue expression (≥ 50%) [4]. The standard method to determine the levels of PD‐L1 is immunohistochemistry (IHC) of tumour tissues, which is recommended for all patients with newly diagnosed advanced NSCLC in routine clinical practice [5]. However, PD‐L1 expression does not seem to be an optimal predictive biomarker since not all patients experience an effective response to ICIs based on the established selection criteria [6]. Thus, the addition of pembrolizumab to platinum‐based chemotherapy in patients with previously untreated advanced NSCLC has recently produced a significant improvement in survival outcomes, independent of PD‐L1 expression [7, 8].

The determination of PD‐L1 expression in tissues is highly variable according to the time and site of biopsy, and sometimes, PD‐L1 expression is not detected due to the limited tissue sample. Moreover, a unique tissue biopsy may not be representative of the entire molecular landscape of the tumour, and therefore, some PD‐L1‐positive patients do not receive immunotherapy. The management of NSCLC with ICIs requires the identification of new and reliable biomarkers to select patients who will benefit from immunotherapy while limiting ineffective therapy that may produce adverse reactions in patients [9]. Liquid biopsy has emerged as a rapid and noninvasive alternative tool to obtain new biomarkers of several cancers and to monitor its evolution over time [10, 11, 12]. Circulating free DNA (cfDNA) and circulating tumour cells (CTCs) are the most common and standardized liquid biopsy biomarkers, representing promising tools for the diagnosis, selection of ICI treatment and monitoring of patients with NSCLC receiving immunotherapy [13]. Moreover, analyses using combinations of multiple liquid biopsy biomarkers are being conducted to improve the accuracy of detection [14, 15].

Some studies have suggested that monitoring cfDNA dynamics might help clinicians select patients with NSCLC who will benefit most from immunotherapy [15, 16, 17]. In this study, we explored the value of the cfDNA determination to anticipate the evolution of metastatic NSCLC in patients receiving first‐line pembrolizumab as a monotherapy or combination therapy. In addition, we also focused our attention on CTC levels, including an analysis of the PD‐L1‐positive subpopulation, to complete our liquid biopsy approach. For this aim, we compared CTC enrichment technologies, such as an epitope‐dependent, EpCAM‐based system (CellSearch^®^; Menarini, Silicon Biosystems, Bologna, Italy), with an epitope‐independent, microfluidic system (Parsortix; Angle Inc., Guildford, UK). Additionally, PD‐L1 expression was analysed in the enriched CTCs using both technologies. Overall, cfDNA and CTC monitoring provides clinically relevant information to select patients who will benefit most from immunotherapy. To our knowledge, this study is the first to examine the association of combined levels of both circulating biomarkers with survival and the response to first‐line pembrolizumab therapy in patients with metastatic NSCLC.

## 2 Materials and methods

### 2.1 Cell lines and culture

The lung cancer cell lines A549, NCI‐H322 and NCI‐H460 were purchased from the American Type Culture Collection (ATCC^®^ CCL‐185™; Manassas, VA, USA) and routinely cultured in the ATCC‐recommended growth medium at 37 °C, 5% CO_2_ and 95% humidity. Cancer cell lines were treated with 100 ng·mL^−1^ IFN‐γ (Merck KGaA, Darmstadt, Germany) for 48 h to obtain different levels of PD‐L1 expression.

### 2.2 Patients and blood sample collection

**Fig. 1:**
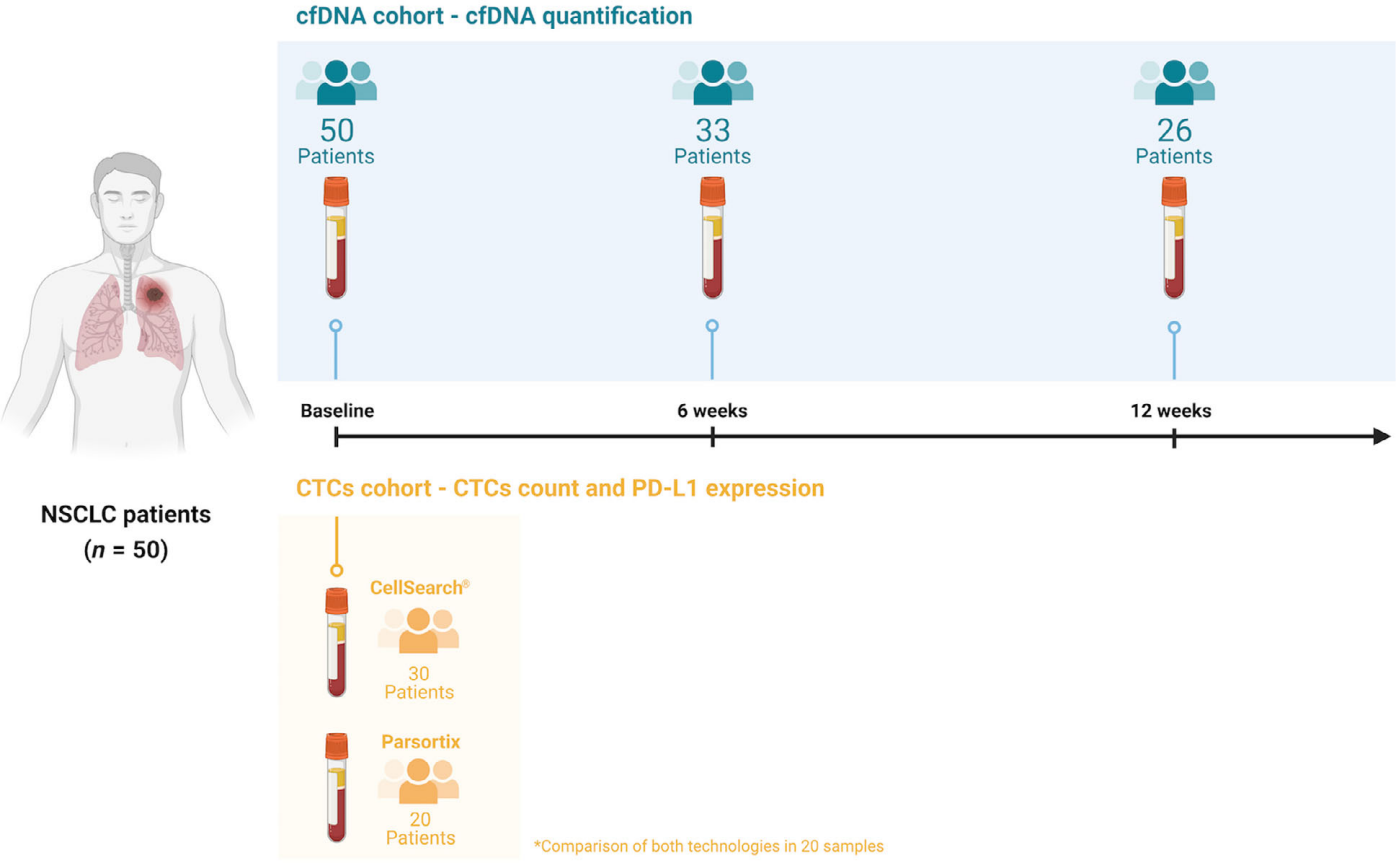
Study schema and monitoring of the patient cohort, including patient enrolment and sample collection.

We designed a prospective study including patients with advanced NSCLC treated with pembrolizumab as first‐line therapy between June 2017 and January 2021 at the Department of Medical Oncology of Complexo Hospitalario Universitario de Santiago de Compostela (Fig. 1). Fifty consecutive patients were recruited. Samples were collected from each patient at different time points: prior to the start of treatment (baseline) and 6 and 12 weeks after the first pembrolizumab dose (Fig. 1). One hundred and nine peripheral blood samples were obtained from patients. All individuals provided written informed consent prior to enrolling in the study, and the procedure was approved by Santiago de Compostela and Lugo Ethics Committee (Ref: 2017/538). The approved protocol was conducted according to the Declaration of Helsinki.

The efficacy of the treatment was evaluated based on RECIST1.1 criteria as follows: complete response (CR), partial response (PR), stable disease (SD) or progressive disease (PD). Progression‐free survival (PFS) was defined as the time from the date of initial treatment until the date of disease progression, death or the last follow‐up if progression or death had not occurred. Overall survival (OS) was defined as the time from the date of initial treatments until death or the last follow‐up.

### 2.3 CfDNA isolation from plasma samples

Twenty millilitres of peripheral whole blood from patients with cancer was obtained by direct venipuncture and collected using CellSave tubes (Menarini, Silicon Biosystems, Bologna, Italy). Plasma was separated within 96 h after blood collection through two sequential centrifugation steps (10 min at 1600 ***g*** and 10 min at 6000 ***g***; both at room temperature) and then stored at −80 °C until further processing. CfDNA was extracted from 3 mL of plasma using a QIAamp Circulating Nucleic Acid Kit (Qiagen, Hilden, Germany) and a vacuum pump, according to the manufacturer’s instructions.

### 2.4 CfDNA quantification

Circulating free DNA yields were determined using the quantitative PCR (qPCR) method by analysing the telomerase reverse transcriptase (*hTERT*) single‐copy gene (Thermo Fisher Scientific, Waltham, MA, USA), as previously reported [18]. The hydrolysis probe is located on chr. 5:1253373 with an 88‐bp amplicon that maps within exon 16 of the *TERT* gene. qPCR was carried out in a final volume of 20 µL consisting of 10 µL of TaqMan Universal Mastermix (Thermo Fisher Scientific), 1 µL of *hTERT* hydrolysis probe and 2 µL of sample. Amplification was performed under the following cycling conditions using a QuantStudio™ 3 real‐time PCR system (Thermo Fisher Scientific): 50 °C for 2 min; 95 °C for 10 min; 40 cycles of 95 °C 15 s; and 60 °C for 1 min. Data were analysed with quantstudio™ Design & Analysis software, version 2.5.1 (Thermo Fisher Scientific).

Each plate included a calibration curve and negative controls. The calibration curve was calculated based on a dilution series of standard human genomic DNA (Roche Diagnostics, Mannheim, Germany) fragmented into 184‐bp fragments using a Covaris^®^ E220‐focused ultrasonicator (Covaris Inc., Woburn, MA, USA). gDNA was fragmented in a 6 × 16 mm microTUBE AFA Fibre Pre‐Slit Snap‐Cap (Covaris) using the following settings: 430‐s duration, peak incident power of 175 Watts, duty factor of 10% and 200 cycles per burst. Fragment sizes were then determined using a TapeStation 4700 (Agilent, Santa Clara, CA, USA) and High Sensitivity DNA ScreenTape^®^ (Agilent). Each sample was analysed in duplicate, and the final concentration was calculated by interpolation of the mean of the quantification cycle (Cq) with the calibration curve. Values with a Cq confidence interval less than 0.95 were discarded. Moreover, only assays with *R* ^2^ values greater than 0.98 for the standard curve and with an efficiency ≥ 88.8% were used.

### 2.5 Spiked experiments

The assays to evaluate PD‐L1 expression on CTCs were tested using the cancer cell lines A549, NCI‐H322 and NCI‐H460 spiked in whole blood from the healthy volunteers recruited for this study. The protocol employed was described previously [12]. Briefly, cells were trypsinized to approximately 80% confluence, and then, 200 cells were added manually (with a calculated pipetting error of 10%) to a total of 7.5 mL of blood from healthy donors collected in CellSave tubes (Menarini, Silicon Biosystems). The samples were analysed using the CellSearch^®^ and Parsortix systems, and two tubes of the same sample were analysed with both technologies. All spiked samples were enriched within 48 h of collection.

### 2.6 Analysis of PD‐L1 expression on CTCs isolated using CellSearch^®^

A total of 7.5 mL of peripheral whole blood samples was collected in CellSave tubes (Menarini, Silicon Biosystems) for CTC enumeration using the CellSearch^®^ system (Menarini, Silicon Biosystems). A CellSearch^®^ CXC Kit (Menarini, Silicon Biosystems) was used for these specific experiments, including ferrofluids coated with epithelial cell‐specific anti‐EpCAM antibodies to immunomagnetically enrich epithelial cells; a mixture of antibodies against cytokeratins (CKs) 8, 18 and 19 conjugated to fluorescein (FLU); an anti‐CD45 mAb conjugated to allophycocyanin (APC); and nuclear dye 4′,6‐diamidino‐2‐phenylindole (DAPI) to fluorescently label the cells. The open 4th antibody position of the CellSearch^®^ system was used to evaluate PD‐L1 expression according to the ‘Guideline for the Use and Optimization of User Defined Markers: CellSearch^®^ Epithelial Cell Kit and CellSearch^®^ CXC Kit, version 1.0’ for its optimization. We employed the anti‐human B7‐H1/PD‐L1 phycoerythrin (PE)‐conjugated antibody (Cat N° FAB1561P; R&D Systems, Minneapolis, MN, USA) at a final concentration of 20 μg·mL^−1^, as described previously [19]. CTCs were identified as EpCAM^+^, CK^+^, CD45^−^ and DAPI^+^, and PD‐L1 expression was recorded for each CTC (presence or absence) by comparison with the PD‐L1 expression levels in the cell lines. The specificity of the staining was confirmed by the lack of signals detected with our negative cell line, A549. As positive control, we employed the cell line NCI‐H460 stimulated with IFN‐γ.

### 2.7 Analysis of PD‐L1 expression on CTCs isolated using the Parsortix system

A total of 7.5 mL of peripheral whole blood was collected in CellSave tubes (Menarini, Silicon Biosystems) and loaded into a Parsortix microfluidic device (Angle Inc., Guildford, UK), as described previously [12]. Briefly, CTCs were then enriched from blood samples in disposable Parsortix cassettes with a size of 6.5 μm (GEN3D6.5; Angle Inc., Guildford, UK) and at 99 mbar of pressure, according to the manufacturer’s guidelines. CTCs were trapped in the Parsortix cassette due to their large size and lower compressibility than the remaining blood cells. After separation, we fixed the sample with 4% paraformaldehyde and carried out on‐cassette staining with selected antibodies according to the manufacturer’s guidelines, followed by fluorescence microscopy detection (Leica DMI8; Leica Microsystems, Wetzlar, Germany). The selected antibodies included Alexa Fluor 647‐conjugated CD45 (35‐Z6, sc‐1178; Santa Cruz, CA, USA) at a final concentration of 4 μg·mL^−1^ to detect white blood cells; the same anti‐human B7‐H1/PD‐L1 PE‐conjugated antibody (R&D Systems) used in CellSearch^®^ systems at a final concentration of 20 μg·mL^−1^ to detect PD‐L1 expression; Alexa Fluor 488‐conjugated pan‐CK (A4‐108‐C100; EXBIO Praha, Vestec, Czech Republic) at a concentration of 1.33 μg·mL^−1^; and DAPI to fluorescently label the cells. CTCs were identified as CK+, CD45‐ and DAPI+, and PD‐L1 expression on CTCs was determined (presence or absence) according to the results obtained from cell line‐spiked samples. We employed Leica Application Suite X (LAS X) (Leica Microsystems) to identify the fluorescence intensity in each single cell, and PD‐L1 expression on CTCs was determined (presence or absence) according to the results obtained from cell line‐spiked samples. The specificity of the staining was confirmed by the lack of signals detected with our negative cell line, A549. As positive control, we employed the cell line NCI‐H460 stimulated with IFN‐γ.

### 2.8 PD‐L1 immunohistochemistry and scoring

PD‐L1 IHC was carried out on 4‐μm sections of FFPE tumour tissue samples using Dako PD‐L1 IHC 28‐8 PharmaDx (Agilent). The test was performed using the EnVision FLEX visualization system on the Dako Autostainer Link 48 and Dako PT Link Pretreatment Module (Agilent). A minimum of 100 viable tumour cells must be present for evaluation. PD‐L1 expression was evaluated only in tumour cells. Scoring was determined according to the tumour proportion score (TPS), which is defined as the percentage of positive viable tumour cells among all viable tumour cells evaluated. A tumour cell was defined as positive for PD‐L1 staining whenever any partial or complete membranous staining was detected. The percentage of PD‐L1‐positive tumour cells was assessed as previously described [20]. Slides were assessed independently by two pathologists.

### 2.9 Statistical analysis

Statistical analyses were performed using r version 4.0.2. The Spearman correlation coefficients were calculated to assess the correlation between the PD‐L1 TPS and PD‐L1 status of CTCs. The kappa test was used to determine the concordance with a 95% confidence interval (CI). We dichotomized the CTC PD‐L1 counts as positive and negative and categorized PD‐L1 expression (PD‐L1 tissue expression, 80–100% vs < 80%) to calculate the kappa coefficients. Receiver operating characteristic (ROC) curves were constructed, and the area under the ROC curve (AUC) with 95% CIs was obtained to evaluate the thresholds of baseline *hTERT* cfDNA levels for OS and PFS analyses. The AUC and the 95% CIs for the sensitivity and specificity were estimated using the pROC package in r software [21]. Univariate and multivariate Cox regression analyses were performed using the survival package in r [22], and a Kaplan–Meier analysis was then performed. The associations between CTCs and *hTERT* cfDNA with the best response were estimated using Fisher’s exact test. We also used Fisher’s exact test to compare the association between CTC counts and the response to therapy.

## 3 Results

### 3.1 Study population

The characteristics of the patients enrolled in the study are summarized in Table 1. The median age was 63.3 years (range: 45–79), and the majority of patients were males (74%), and current or former smokers (86%) and had tumours with an adenocarcinoma histology (72%). Eighty per cent of patients had an Eastern Cooperative Oncology Group Performance Status (ECOG PS) of 1–2, and 30% of patients had more than two metastases. Thirty‐four per cent of patients exhibited PD‐L1 expression in the tissue at a level ≥ 80%, and the median number of pembrolizumab treatment cycles was 6 (range 1–35 cycles). The median PFS and OS were 10.47 and 19.13 months, respectively, in the 50 patients with NSCLC. The objective response rate (ORR: complete response or partial response during ≥ 6 cycles) was 46.0%, with 1 complete and 22 partial responses.

**Table 1:** Demographics and clinical characteristics of the patients at baseline. SD, standard deviation; NSCLC‐NOS, non‐small‐cell lung cancer‐not otherwise specified.

Baseline characteristics	Patients *n* ^a^ (%)
Mean age (years) ± SD, range	63.3 ± 8.3, 45–79
Sex	
Female	13 (26)
Male	37 (74)
Smoking	
Smoker	31 (62)
Former smoker	12 (24)
Never	7 (14)
Histology	
Adenocarcinoma	44 (88)
Squamous cell carcinoma	4 (8)
NSCLC‐NOS	2 (4)
ECOG PS	
0	10 (20)
1	34 (68)
2	6 (12)
PD‐L1 expression in the tissue	
< 80%	33 (66)
≥ 80%	17 (34)
Number of metastatic sites	
≤ 2	35 (70)
> 2	15 (30)
Pembrolizumab treatment	
Monotherapy	37 (74)
In combination with chemotherapy	13 (26)

^a^ *n* = 50 at baseline.

### 3.2 Circulating free DNA analyses

#### 3.2.1 Prognostic and predictive value of cfDNA levels at baseline

**Fig. 2:**
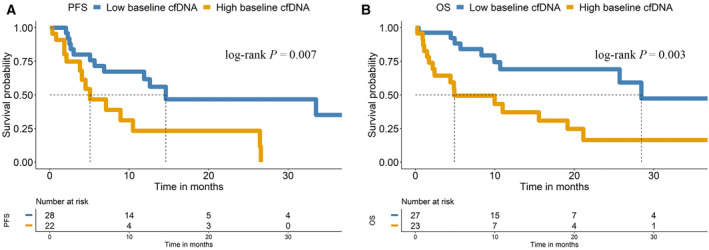
Kaplan–Meier survival analysis of *hTERT* cfDNA levels at baseline. Kaplan–Meier plots of PFS (A) and OS (B).

We next evaluated the role of cfDNA levels as a prognostic biomarker for pembrolizumab treatment outcomes in our cohort of patients with NSCLC. We employed the *hTERT* qPCR assay to determine the cfDNA levels. The cohort was dichotomized into two groups (high and low levels) according to a threshold calculated based on the baseline *hTERT* cfDNA levels observed in our cohort (Table S1). These levels were log10‐transformed by choosing 7.665 (2132.39 in genome equivalents·mL^−1^, GE·mL^−1^ plasma) and 7.638 (2075.59 in genome equivalents·mL^−1^, GE·mL^−1^ plasma) for PFS and OS analyses, respectively, after considering sensitivity and specificity based on ROC curve analyses (Table S1). Patients with high baseline *hTERT* cfDNA levels had a significantly shorter PFS (*P*‐value < 0.01; hazard ratio, 2.89; 95% CI, 1.30–6.45) and OS (*P*‐value = 0.005; hazard ratio, 3.26; 95% CI, 1.43–7.47) than those with low baseline levels (Fig. 2A,B and Table 2).

**Table 2:** Univariate and multivariate Cox regression analyses of cfDNA levels, CTC counts and clinical parameters. ECOG, Eastern Cooperative Oncology Group Performance Score. The levels of cfDNA were determined as low (< cut‐off) or high (≥ cut‐off) based on the cut‐off obtained from the ROC curve analyses.

Variable	Univariate		Multivariate	
	*P*‐value	Hazard ratio (95% CI)	*P*‐value	Hazard ratio (95% CI)
PFS				
Baseline log cfDNA (high vs. low cfDNA, *n* = 50)	0.009	2.89 (1.30–6.45)	0.80	1.18 (0.28–5.01)
Baseline CTC count, CellSearch (≥ 1 vs. 0, *n* = 30)	0.04	2.97 (1.04–8.45)	0.006	9.36 (1.88–46.6)
ECOG (≥ 1 vs. 0, *n* = 50)	0.20	2.09 (0.72–6.08)	0.70	1.40 (0.24–8.15)
PD‐L1 expression in the tissue (≥ 80 vs. < 80, *n* = 50)	0.40	0.68 (0.27–1.76)	0.13	0.37 (0.10–1.36)
Sex (male vs. female, *n* = 50)	0.40	0.71 (0.32–1.57)	0.04	0.23 (0.05–0.97)
Age (years, *n* = 50)	0.60	0.99 (0.93–1.04)	0.30	1.05 (0.96–1.16)
Number of metastasis (> 2 vs. ≤ 2, *n* = 50)	0.04	2.39 (1.03–5.55)	0.006	9.21 (1.87–45.3)
Smoking (yes vs. no, *n* = 50)	0.40	0.68 (0.25–1.82)	0.03	11.4 (1.25–104)
Combined changes in CTC and cfDNA levels^a^				
Group 1 (CTCs < 1 and a low cfDNA level, *n* = 12)	–	Reference	–	–
Group 2 (CTCs < 1 and a high cfDNA level or CTCs ≥ 1 and a low cfDNA level, *n* = 10)	0.005	5.37 (1.66–17.4)	0.009	13.1 (1.91–90.1)
Group 3 (CTCs ≥ 1 and a high cfDNA level, *n* = 8)	0.05	4.12 (0.98–17.4)	0.01	14.5 (1.76–119)
Combined changes in CTC and cfDNA levels^a^				
Group A (CTCs < 1 and a low cfDNA level, *n* = 12)	–	Reference	–	–
Group B (CTCs < 1 and a high cfDNA level or CTCs ≥ 1 and a low cfDNA level or CTCs ≥ 1 and a high cfDNA level, *n* = 18)	0.006	4.99 (1.60–15.6)	0.005	13.6 (2.17–85.3)
OS				
Baseline log cfDNA (high vs. low cfDNA levels, *n* = 50)	0.005	3.26 (1.43–7.47)	0.90	1.13 (0.29–4.46)
Baseline CTC count, CellSearch (≥ 1 vs. 0, *n* = 30)	0.03	2.71 (1.11–6.64)	0.01	5.41(1.42–20.6)
ECOG (≥ 1 vs. 0, *n* = 42)	0.06	4.05 (0.95–17.2)	0.70	1.51 (0.25–8.99)
PD‐L1 expression in the tissue (≥ 80 vs. < 80, *n* = 50)	0.50	1.36 (0.60–3.10)	0.50	0.64 (0.20–2.04)
Sex (male vs. female, *n* = 42)	0.70	1.18 (0.49–2.86)	0.20	0.42 (0.10–1.76)
Age (years, *n* = 42)	0.80	1.01 (0.95–1.07)	0.20	1.07 (0.98–1.17)
Number of metastasis (>2 vs. ≤2, *n* = 42)	0.005	3.12 (1.41–6.87)	0.001	9.08 (2.35–35.1)
Smoking (yes vs. no, *n* = 42)	0.60	0.77 (0.29–2.05)	0.02	20.5 (1.54–273)
Combined changes in CTC and cfDNA levels^a^				
Group 1 (CTCs < 1 and a low cfDNA level, *n* = 12)	–	Reference		
Group 2 (CTCs < 1 and a high cfDNA level or CTCs ≥ 1 and a low cfDNA, *n* = 10)	0.14	2.33 (0.76–7.15)	0.50	1.90 (0.33–11.0)
Group 3 (CTCs ≥ 1 and a high cfDNA level, *n* = 8)	0.02	4.24 (1.29–14.0)	0.01	6.39 (1.37–29.8)
Combined changes in CTC and cfDNA levels^a^				
Group A (CTCs < 1 and a low cfDNA level, *n* = 12)	–	Reference	–	–
Group B (CTCs < 1 and a high cfDNA level or CTCs ≥ 1 and a low cfDNA level or CTCs ≥ 1 and a high cfDNA level, *n* = 18)	0.04	2.91 (1.05–8.07)	0.05	4.02 (0.97–16.6)

^a^ Multivariate Cox regression model including sex, age, Eastern Cooperative Oncology Group Performance Score, PD‐L1 expression in the tissue, number of metastases and smoking status.

The median OS was 28.4 months in the low baseline *hTERT* cfDNA group and 4.9 months in the high baseline *hTERT* cfDNA group, whereas the median PFS was 14.6 and 5.1 months in the two cfDNA categories (low vs. high baseline levels, respectively) (Fig. 2A,B). Considering various clinical and demographic variables (ECOG PS, sex, age, PD‐L1 expression in the tissue, number of metastases and smoking status), univariate and multivariate Cox regression analyses of PFS and OS were performed (Table 2). In this analysis, *hTERT* cfDNA levels did not show value as an independent predictive biomarker of PFS and OS, with the number of metastases representing the main independent factor explaining the PFS rates.

#### 3.2.2 Monitoring hTERT cfDNA levels and the response to therapy

**Fig. 3:**
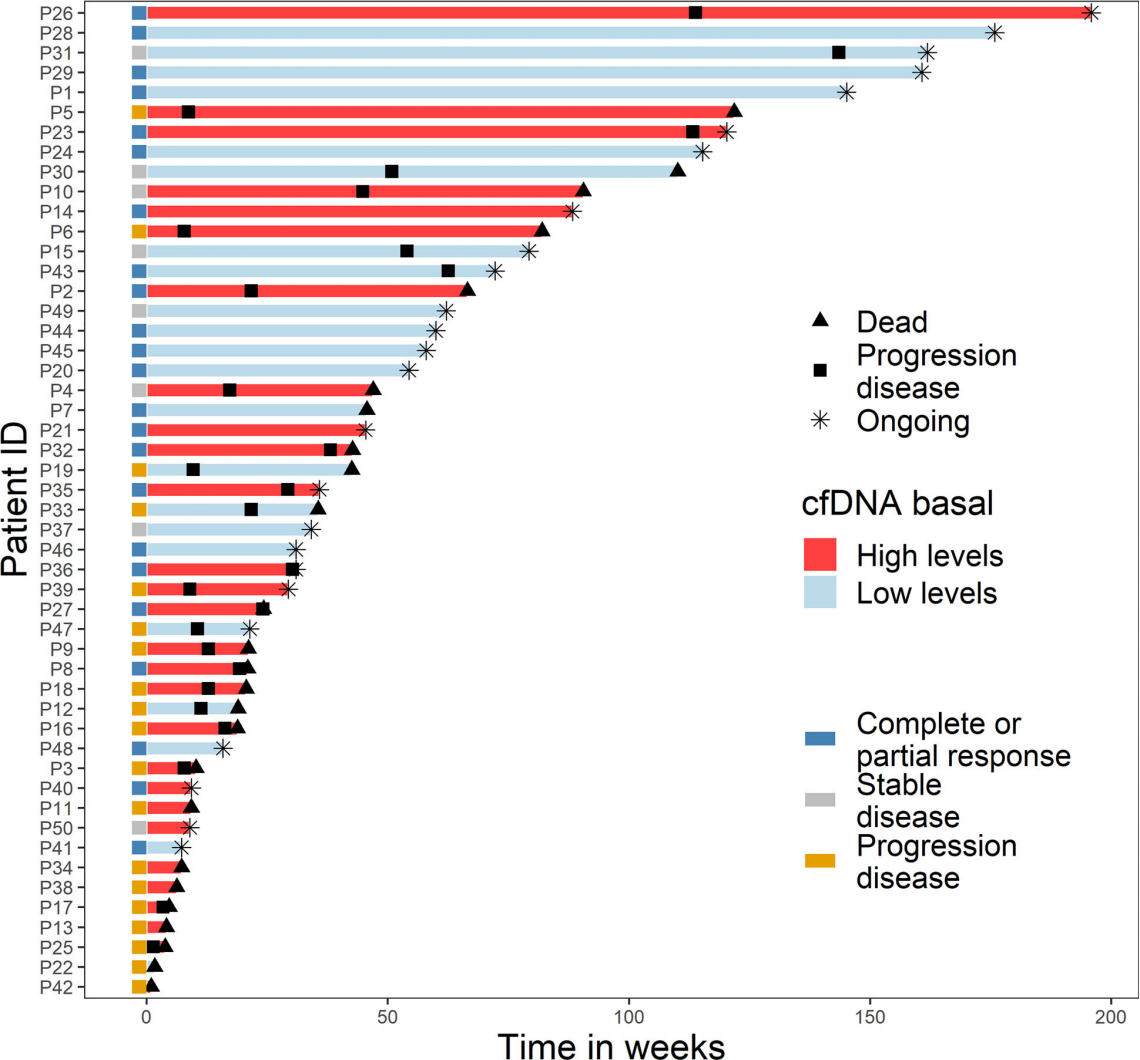
Clinical course for patients during pembrolizumab treatment. Swimmer plots for each patient (*n* = 50) showing the levels of *hTERT* cfDNA at baseline (red colour indicates high *hTERT* cfDNA levels, and blue colour indicates low *hTERT* cfDNA levels). The total length of each bar indicates the duration of survival from start of pembrolizumab treatment. Left, squares are coloured according to the response based on RECIST1.1 criteria.

**Fig. 4:**
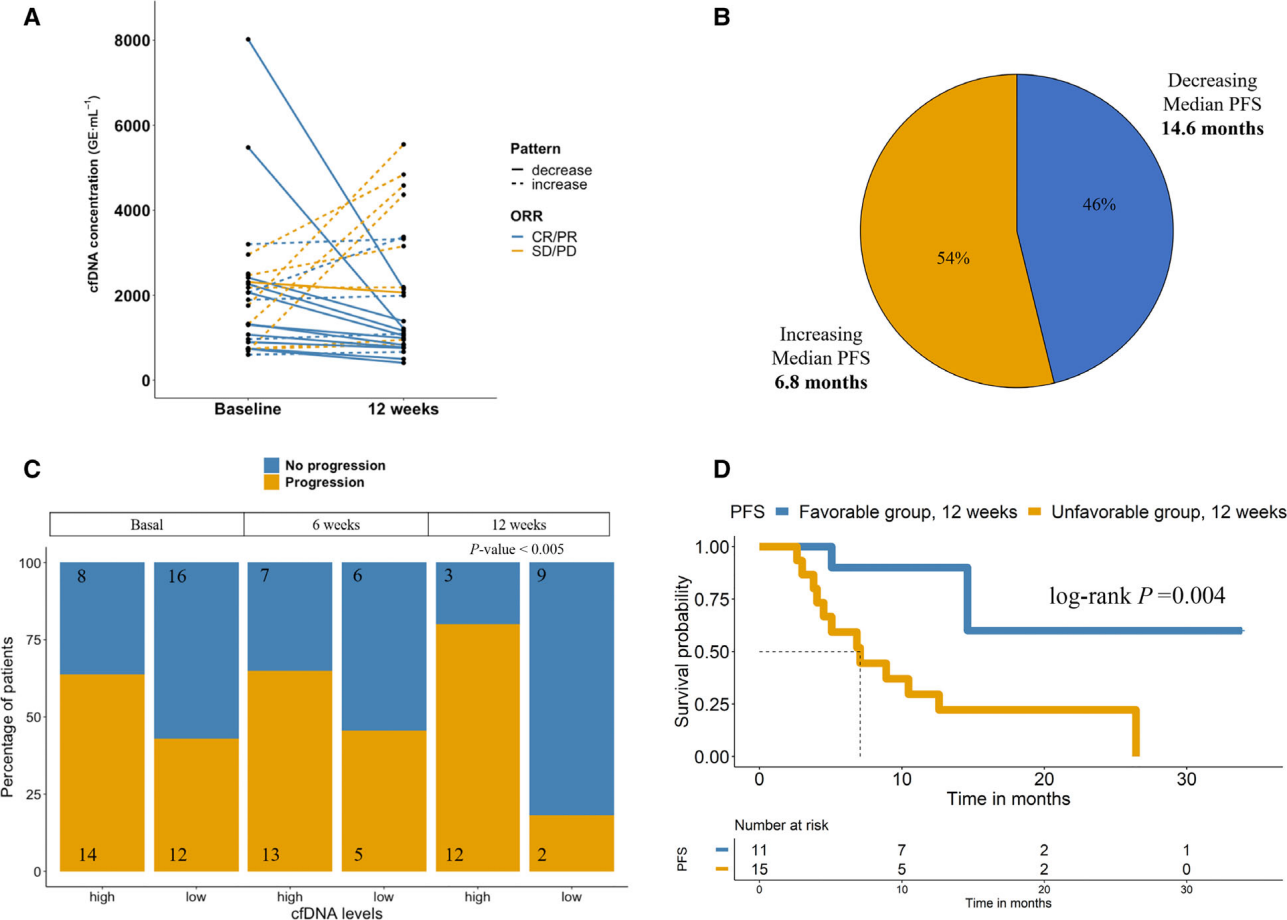
*hTERT* cfDNA changes from baseline to 12 weeks. (A) *hTERT* cfDNA concentrations for the two cfDNA patterns (increase/decrease at 12 weeks) and showing the response to therapy. (B) Percentage of patients and median PFS for each cfDNA pattern; (C) Proportion of patients with high and low levels baseline, 6 and 12 weeks. *P*‐value was calculated by Fisher’s exact test. (D) Kaplan–Meier plot of PFS of the favourable/unfavourable changes at 12 weeks. Groups are composed of patients with unfavourable changes: both high *hTERT* cfDNA levels at baseline (cut‐off ≥ 7.665) and at 12 weeks (cut‐off ≥ 7.026), or low levels at baseline (cut‐off < 7.665) and high levels at 12 weeks (cut‐off ≥ 7.026). The low‐risk group (patients with favourable changes) is composed of patients with low *hTERT* cfDNA levels at baseline (cut‐off < 7.665) and low levels at 12 weeks (cut‐off < 7.026) or high levels at baseline (cut‐off ≥ 7.665) and low levels at 12 weeks (cut‐off < 7.026). CR/PR, complete response/partial response; SD/PD, stable disease/progression disease.

We next investigated the value of *hTERT* cfDNA kinetics as a prognostic biomarker during pembrolizumab treatment (Figs 3 and 4). Blood samples were collected longitudinally, before the initiation of therapy, and at 6 and 12 weeks after the onset of pembrolizumab therapy (Fig. 1). No significant differences were observed in the global cfDNA levels between any group (Fig. S1A). We monitored and investigated the relationship between *hTERT* cfDNA levels and the response to therapy in our patient cohort (Fig. 3). We did not find any association between the cfDNA levels at baseline and the response to pembrolizumab therapy (Fig. S1B). However, after considering the changes from baseline to 12 weeks, we found an association with treatment response (Fig. 4A). We observed two patterns: an increase in *hTERT* cfDNA levels at 12 weeks (*n* = 14) and a decrease in *hTERT* cfDNA levels at 12 weeks (*n* = 12), with a median PFS of 6.8 and 4.6 months, respectively (Fig. 4B).

We also analysed the risk of disease progression at each time point. According to the ROC curve analysis, the thresholds of cfDNA levels at baseline, 6 weeks and at 12 weeks were chosen for the PFS analysis (Table S1). High levels of *hTERT* cfDNA at 12 weeks were a strong predictor of the risk of disease progression (*P*‐value < 0.005, odds ratio = 18, 95% CI 2.5–131.3) (Fig. 4C). Fifteen patients showed high levels at 12 weeks, and 12 of them (80%) developed progressive disease compared with 2 of the 11 patients (18.2%) with low levels. Moreover, at each time point, patients were divided into favourable and unfavourable risk groups after considering their changes in *hTERT* cfDNA levels. The median PFS was 7.07 months for the unfavourable risk group based on the changes between baseline and 12 weeks, whereas median PFS was not reached for the favourable risk group (*P*‐value < 0.01; hazard ratio, 6.8; 95% CI, 1.5–30.5) (Fig. 4D).

### 3.3 Circulating tumour cell analyses

#### 3.3.1 EpCAM‐dependent versus antigen‐independent CTC isolation to quantify CTCs and characterize the PD‐L1 status

**Fig. 5:**
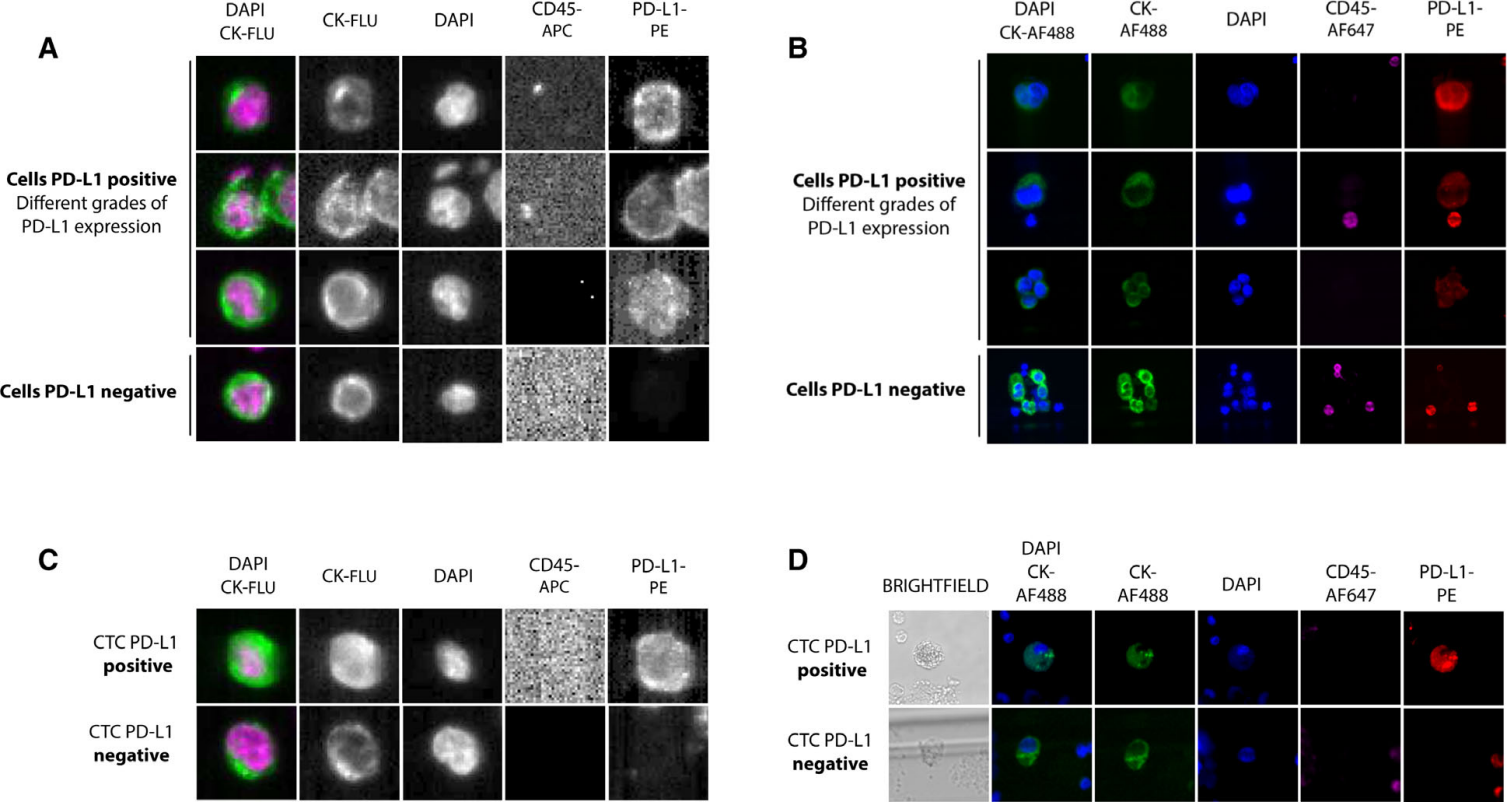
(A, B) Detection of PD‐L1 expression after spiking cancer cell lines in healthy blood and analysis of them with the CellSearch^®^ and Parsortix systems, respectively, and representative images of different grades of PD‐L1 expression. (C) Representative images of CTCs detected with the CellSearch^®^ system in patients with NSCLC. Samples were subjected to immunostaining with DAPI, CD45 (APC), cytokeratins (FLU) and PD‐L1 (PE). (D) Representative images of CTCs detected with the Parsortix system in patients with NSCLC. Samples were subjected to immunostaining with DAPI, CD45 (AF647), cytokeratins (AF488) and PD‐L1 (PE). NCI‐H460 stimulated with IFN‐γ shown high expression, NCI‐H460, medium expression, NCI‐H322, low–medium expression, and A549, no expression.

In addition to monitoring *hTERT* cfDNA levels, we analysed CTC levels, as they represent a more biological feature of the tumour. Therefore, we evaluated two different technologies, the EpCAM‐based CellSearch^®^ system and the label‐independent microfluidic Parsortix system, to quantify CTCs and assess the expression of PD‐L1 and to determine the advantages of the non‐EpCAM‐dependent isolation method. First, we categorized the presence or absence of PD‐L1 on single CTCs to grade its expression in each cell line (with known gradual increases in PD‐L1 protein expression) using both approaches (Fig. 5A,B). We used preserved blood samples from healthy controls spiked with three lung cancer cell lines (A549, *no expression*; NCI‐H322, *low‐medium expression*; and NCI‐H460, *medium‐high expression*) representative of the variability of PD‐L1 expression (Fig. S2).

Next, peripheral blood samples were collected from patients prior to treatment to analyse the presence of CTCs and their PD‐L1 expression (Fig. 5C,D) as potentially valuable prognostic and predictive biomarkers. In 20 samples from patients, we compared CTC enumeration using both strategies (CellSearch^®^ and Parsortix systems) and evaluated the performance and concordance between them (Fig. S3A,B and Table S2). Using the CellSearch^®^ system, we detected ≥ 1 CTC in 50% (10/20) of samples (range 1–168; mean = 9.8), while using the Parsortix system, 35% (7/20) of samples had ≥ 1 CTC (range 1–56; mean = 4.7). Regarding the capacity to detect PD‐L1 expression in CTCs, we detected PD‐L1‐positive CTCs in 2 of 17 samples (in 2/10 samples with CTCs) using the CellSearch^®^ system. Compared with the label‐independent system, we observed PD‐L1‐positive CTCs in 7 of 20 samples (in 7/7 samples with CTCs) using the Parsortix system. When we compared the concordance of both technologies, we found that kappa scores for the number of CTCs and PD‐L1‐positive CTCs presented negative values for both technologies (−0.1 and 0.14, respectively), showing no correlation between them.

#### 3.3.2 Correlation of the PD‐L1 status in CTCs and tissue samples

We also compared the PD‐L1 status of CTCs using CellSearch^®^ and Parsortix systems with PD‐L1 expression in the primary tumour biopsy obtained at the initial diagnosis (Fig. S4). The PD‐L1 TPS in biopsies did not correlate with the percentage of PD‐L1‐positive CTCs at the initial diagnosis (*P*‐value = 0.59 and *P*‐value = 0.71 for the CellSearch^®^ and Parsortix systems, respectively). The mean time between tissue biopsy and liquid biopsy sample collection was 38.6 days (range 9–78). Kappa scores for the PD‐L1 status in CTCs and PD‐L1 expression in tissue were low for both technologies. Thus, using the CellSearch^®^ system, the concordance rate was 26.7% with Cohen’s kappa of 0.04, while with the Parsortix system, the concordance rate was higher, 47.1% with Cohen’s kappa of 0.14.

#### 3.3.3 Prognostic and predictive values of CTC enumeration at baseline

**Fig. 6:**
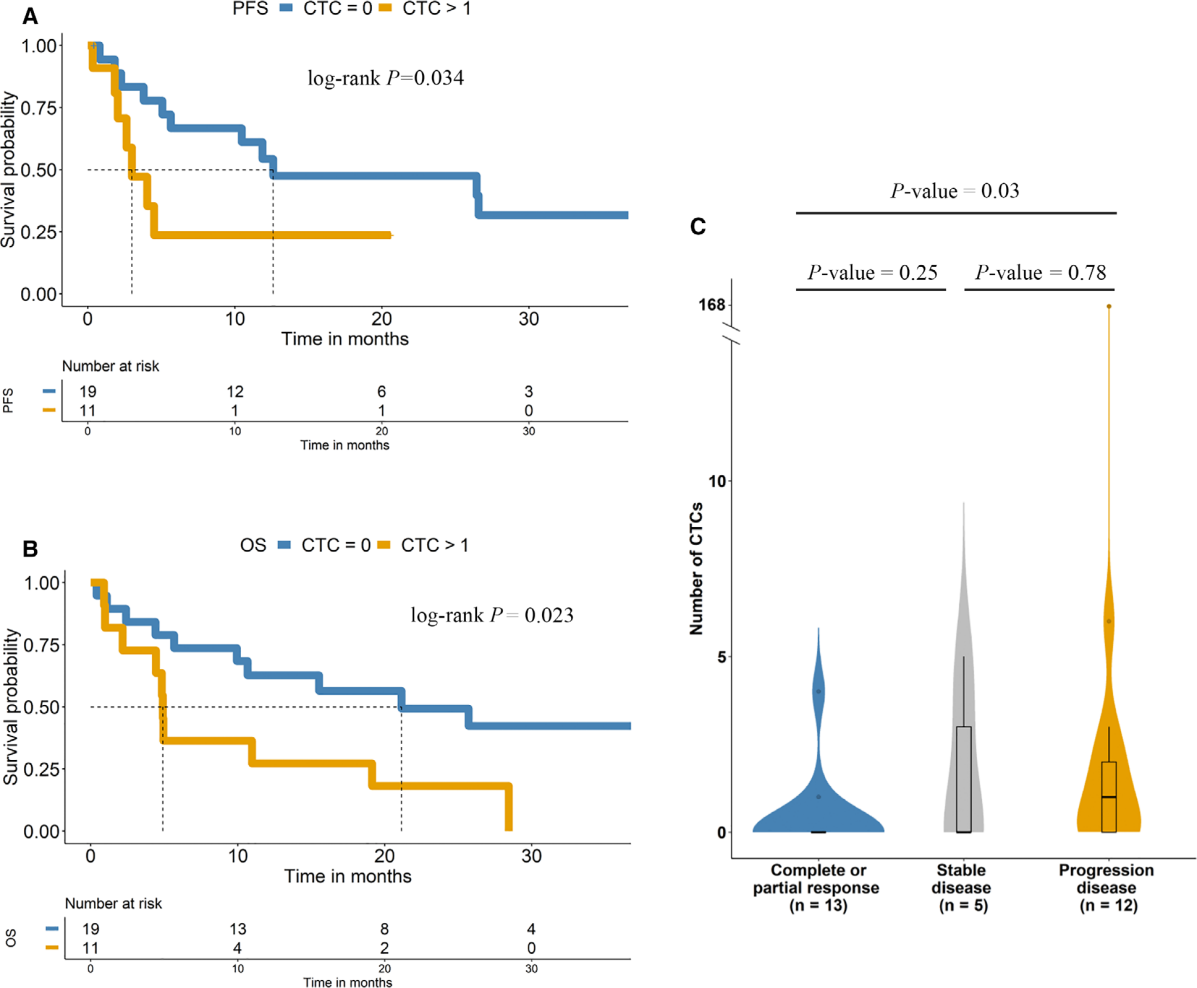
Kaplan–Meier survival analysis of CTCs at baseline. Kaplan–Meier plots of PFS (A) and OS (B). (C) Comparison of the response to pembrolizumab based on CTC detection using CellSearch^®^. *P*‐value was calculated by Fisher’s exact test.

We evaluated the role of CTC enumeration in prognosticating disease progression in response to pembrolizumab treatment in our cohort of patients with advanced NSCLC. For this purpose, we performed CTC enumeration at baseline in 30 patients using the CellSearch^®^ system and 20 patients using Parsortix. Patients with CTCs identified using the CellSearch^®^ system had significantly shorter PFS and OS than patients who had no CTCs (*P*‐value < 0.05) (Fig. 6A,B). The median PFS was 12.6 and 3 months in the two groups (0 vs. ≥ 1 CTC, respectively). The median OS of the CTC‐positive group was 4.9 months, whereas the median OS was 21.13 months for the CTC‐negative group. In the multivariate regression analysis (Table 2), we confirmed that CTC positivity using the CellSearch^®^ system was an independent predictive biomarker of PFS and OS (hazard ratio, 5.75; 95% CI, 1.35–24.5, *P*‐value < 0.05 and hazard ratio, 4.59; 95% CI, 1.32–16.0, *P*‐value < 0.05, respectively). We observed no significant results for PFS or OS when analysing CTC counts with the Parsortix system and evaluating PD‐L1‐positive CTCs, regardless of the technology employed (CellSearch^®^ and Parsortix systems).

We also investigated the relationship between the CTC counts detected using both approaches and the response to pembrolizumab therapy in our patient cohort. Significant differences were observed between the CTC count detected with the CellSearch^®^ system and the achievement of a complete or partial response and disease progression (*P*‐value < 0.05) (Fig. 6C). The ORR was similar between patients with undetectable CTCs compared with patients with detectable PD‐L1‐positive or PD‐L1‐negative CTCs using CellSearch^®^ and Parsortix technologies (Table S3).

### 3.4 Clinical potential of combined CTC and cfDNA analyses

We also evaluated the joint effect of baseline CTC counts using the CellSearch^®^ system and *hTERT* cfDNA levels. First, we considered three risk subgroups: (a) CTCs < 1 and low cfDNA baseline levels; (b) CTCs < 1 and high cfDNA baseline levels or CTCs ≥ 1 and low cfDNA baseline levels; and (c) CTCs ≥ 1 and high cfDNA baseline levels. Multivariate Cox analyses confirmed that the combined analyses of CTCs using the CellSearch^®^ system and *hTERT* cfDNA levels were independent predictive biomarkers of PFS (*P*‐value < 0.01; hazard ratio, 11.6; 95% CI, 2.04–66.1; *P*‐value < 0.05; hazard ratio, 14.3; 95% CI, 1.7–117) (Table 2).

**Fig. 7:**
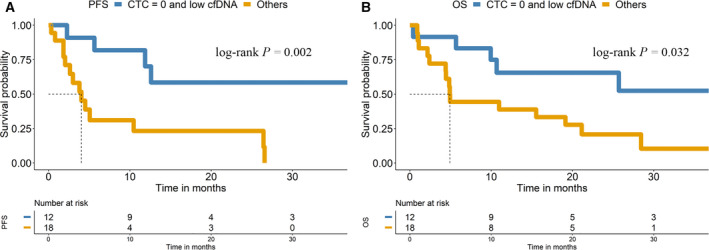
CTCs and *hTERT* cfDNA correlate with the prognosis of patients with NSCLC treated with pembrolizumab. (A) Kaplan–Meier survival plot of PFS based on the combination of cfDNA and CTC levels at baseline. (B) Kaplan–Meier survival plot of OS based on the combination of cfDNA and CTC levels at baseline.

Subsequently, we considered only two risk subgroups to simplify the analysis: (A) CTCs < 1 and low cfDNA baseline levels; and (B) CTCs < 1 and high cfDNA baseline levels or CTCs ≥ 1 and low cfDNA baseline levels or CTCs ≥ 1 and high cfDNA baseline levels. Patients from subgroup A (undetectable CTCs using CellSearch^®^ and low cfDNA levels) had significantly shorter PFS (*P*‐value < 0.01; hazard ratio, 4.99; 95% CI, 1.6–15.6) and OS (*P*‐value < 0.05; hazard ratio, 2.9; 95% CI, 1.1–8.1) than patients from subgroup B (Table 2). The median PFS was not reached in group A (low cfDNA levels and undetectable CTCs at baseline), whereas the median PFS was 4.0 months in group B. Similarly, the median OS was not reached in group A, whereas the median OS was 4.9 months in group B (Fig. 7A,B). Overall, the hazard ratios and *P*‐values that emerged from the univariate and multivariate Cox analyses suggest a combinatory effect of both markers as early predictors of disease progression (Table 2).

Additionally, patients with CTCs identified using the CellSearch^®^ system and high cfDNA levels at baseline showed a trend towards a poorer response to pembrolizumab therapy (Fig. S5).

## 4 Discussion

Liquid biopsy represents a promising tool for the diagnosis, selection and monitoring of response to ICI treatment in the context of NSCLC. Currently, PD‐L1 is the best studied biomarker for ICI treatment selection, exhibiting a higher probability of response with higher expression of PD‐L1 [13]. In patients with advanced NSCLC and PD‐L1 expression on at least 50% of tumour cells, pembrolizumab results in significantly longer PFS and OS [4], and thus, the determination of PD‐L1 expression in tissue samples is the reference factor for the selection of ICI treatments. However, in clinical practice, patients with high levels of PD‐L1 may not respond to ICI treatment; in contrast, in the absence of PD‐L1, a clinical benefit may be obtained from the use of PD‐1 or PD‐L1 checkpoint inhibitors [13]. A high tumour mutational burden (TMB), which is associated with high levels of neoantigens, represents another potential candidate biomarker that would drive the choice of treatment [4], although this biomarker has not been translated into the clinic due to conflicting results among studies [23, 24, 25, 26, 27, 28]. Notably, these two markers have been mainly examined in tissue samples.

In the present study, we analysed the value of *hTERT* cfDNA and CTC analyses as potential prognostic and predictive noninvasive biomarkers to discriminate patients who will benefit from ICIs. We explored the clinical value of monitoring *hTERT* cfDNA levels and detected the presence of CTCs, including PD‐L1 characterization, in a homogeneous cohort of patients with metastatic NSCLC receiving first‐line treatment with pembrolizumab as a monotherapy or in combination with chemotherapy.

Circulating free DNA has already been proposed as a prognostic and predictive biomarker in NSCLC [29, 30, 31], but few studies have reported the cfDNA concentration as a predictive marker of the immunotherapy response. Although cfDNA represents promising material, standardized protocols for the processing and total quantification are still missing [32]. In our work, we propose the qPCR method to quantify *hTERT* cfDNA because it is a sensitivity and cost‐effective assay and based on previous results obtained in lung cancer patients [15, 18, 33]. Alama *et al*. [15] reported that patients with NSCLC who were treated with a 2nd or higher line of nivolumab and with cfDNA level below their cohort median values survived significantly longer than those with a cfDNA level above this threshold. In another study, patients with NSCLC treated mainly with second‐line therapy with nivolumab and with low ctDNA concentrations at the first evaluation showed a long‐term benefit [16]. Notably, numerous studies have described the presence of somatic mutations in plasma cfDNA and their association with tumour response and survival [34, 35, 36]. Recently, an early decrease or total clearance of ctDNA levels after pembrolizumab administration identified subsets of patients with advanced solid tumours who had a good prognosis [37, 38], regardless of the tumour type, PD‐L1 status or TMB. In a previous study, changes in the ctDNA concentration were reported as a predictor of a durable response in patients treated with anti‐PD‐1 drugs [39], where the persistence of ctDNA exerted a detrimental effect. Goldberg *et al*. [40] also suggested that cfDNA levels may be an early marker of therapeutic efficacy, predicting prolonged survival in patients treated with ICIs for NSCLC. Based on these findings, serial ctDNA analyses could serve as a generalizable monitoring strategy for patients treated with ICIs, but researchers have not clearly determined whether this approach is transferable to cfDNA, which is clearly easier to analyse.

Regarding the on‐treatment cfDNA levels, data are scarce, although cfDNA levels appear to be decreased in response to effective treatments [41, 42, 43]. Our study prospectively evaluated the value of *hTERT* cfDNA kinetics as a prognostic biomarker during pembrolizumab treatment. Patients with NSCLC presenting high *hTERT* cfDNA levels at baseline or unfavourable changes from baseline to 12 weeks had a significantly greater risk of disease progression. Importantly, although the concentration of cfDNA can vary among individuals, depending on physiological factors and tumour characteristics [44, 45], the cfDNA analysis has clear advantages compared with the ctDNA determination, such as its feasibility, low detection cost and reproducibility. Therefore, our results reinforced the potential to monitor *hTERT* cfDNA dynamics in patients with NSCLC treated with ICIs, providing a simple tool to better anticipate the response to treatment.

We also investigated CTC levels using two CTC enrichment technologies, the EpCAM‐based CellSearch^®^ system (approved by the Food and Drug Administration (FDA) for the prognostic assessment of CTCs in patients with metastatic breast, colon and prostate cancer [46, 47, 48]) and the microfluidic epitope‐independent‐based method Parsortix system to complete our liquid biopsy approach. The CellSearch^®^ system is the only FDA‐approved device for CTC enumeration in some cancer types, although other immunomagnetic‐based strategies have been developed for CTC enrichment [49, 50]. The Parsortix system uses a combination of size‐based and microfluidic‐based enrichment approaches to separate CTCs from blood samples and solve the dependence on a single biomarker, EpCAM, in this case, allowing the detection of CTCs with a more mesenchymal phenotype. In our study, the detection rate using the CellSearch^®^ system (50%) was higher than that using the Parsortix system (35%), and many differences at the individual level were observed when comparing the results of both strategies for the same patient, reinforcing the isolation of different CTC types with both technologies.

In a previous comparative study of patients with NSCLC, Janning *et al*. [51] reported a higher detection rate using the Parsortix than the CellSearch^®^ system in patients with NSCLC receiving different therapy regimens, attributing the difference to the heterogeneity and low EpCAM expression of some CTCs and therefore to the inability of EpCAM‐based CellSearch^®^ to detect certain subpopulations. Our cohort was a more homogeneous cohort, with patients naive to previous treatments. This last factor can favour the epithelial characteristics of CTCs, since the EMT is induced as a result of drug resistance in NSCLC [52, 53, 54, 55]. However, we analysed only CK‐positive CTCs in combination with PD‐L1; therefore, we were unable to make a firm statement in this regard. Although previous works showed good recovery rates in cell lines and patients using Parsortix system [12, 51, 56], some methodological aspects can be impacting on the CTC enumeration in our cohort. For instance, we used CellSave Preservative tubes, which contain fixative reagents that can modify the deformability properties of the cells. Besides the staining protocol differs from previous publications [51] that described a higher detection rate using the Parsortix system in NSCLC.

Among the CTC population, we focused our attention on the PD‐L1‐positive subpopulation, which represents treatment targets. Although our data show that the determination of the PD‐L1 status is feasible in CTCs from patients with NSCLC, we found that the PD‐L1 status of CTCs does not correlate with the PD‐L1 expression characterized in tissue samples or with the response to the treatment, regardless of the method employed. Despite the lack of prognostic value of PD‐L1 expression on CTCs obtained using both approaches, most of the CTCs isolated using the Parsortix system were PD‐L1‐positive (100% of patients with CTCs), while only 20% of samples with CTCs contained PD‐L1‐positive CTCs using the CellSearch^®^ system. Thus, the epitope‐independent‐based Parsortix system showed a high recovery rate of PD‐L1‐positive CTCs, probably associated with a more mesenchymal phenotype of CTCs isolated with this EpCAM independent strategy. The use of CTCs and their potential to analyse PD‐L1 expression has already been reported in patients with NSCLC [51, 57, 58, 59, 60, 61, 62, 63], including the study by Janning, but their significance is not yet clear [64]. The lack of concordance and contradictory results between the presence of PD‐L1 in tissue and the percentage of PD‐L1‐positive CTCs have also been reported [51, 57, 58, 61, 63, 65]. Importantly, in most studies assessing PD‐L1 expression in CTCs, different antibodies and CTC enrichment technologies have been used, which might partially explain the discrepancy.

Another important point is that CTCs originate from different tumour locations with different PD‐L1 patterns [66], and tissue comparisons have been performed mainly with the primary tumour. Importantly, in our study, no association of PD‐L1 expression on CTCs, such as prognostic or predictive biomarkers, was found. Despite the lack of clinical impact found for PD‐L1‐positive CTCs, the global CTC count at baseline determined using the CellSearch^®^ system was significantly associated with PFS and OS, as previously described in patients treated with chemotherapy [67] and in patients receiving ICI treatment using the CellSearch^®^ system or other technologies [13]. Our results revealed a greater effect of the main epithelial circulating population in patients with NSCLC, since the CTC count determined using a non‐EpCAM‐dependent strategy failed to show any association with the patients’ outcomes.

Finally, our study represents a pioneering approach combining CTC count and cfDNA levels to predict the response of patients with NSCLC to first‐line pembrolizumab treatment. We observed that patients with NSCLC presenting ≥ 1 CTC detected with the CellSearch^®^ system and high levels of *hTERT* cfDNA at baseline had a significantly higher risk of disease progression during pembrolizumab treatment. Our results are consistent with a previous report focused on the prognostic role of these two easy‐to‐measure biomarkers in patients with metastatic NSCLC receiving nivolumab [15]. These results confirmed the value of combining different circulating biomarkers to reach a higher prognostic and predictive accuracy and better discriminate the patients who will benefit most from ICI treatment, in addition to PD‐L1 status.

On the other hand, several limitations in our design should be considered, which precludes us from drawing solid conclusions. First, we used different antibodies to analyse PD‐L1 expression in CTCs and tumours, since the standard procedure was applied in tissue samples, while we used another antibody with CellSearch^®^ and Parsortix. Second, we did not perform CTC monitoring during therapy in our NSCLC cohort, which could provide more valuable information. Third, *hTERT* amplification has been reported in different cancer types, including lung cancer [68, 69]. Although the percentage of NSCLC patients with this alteration is very low (around 5–10% of lung cancer patients), we cannot exclude an overestimation of the cfDNA content in a low percentage of the cases analysed due to a potential amplification. In addition, the sample size of the combined cohort was relatively small.

## 5 Conclusions

In summary, the study served to establish the best strategy to monitor PD‐L1 expression on CTCs from patients with advanced NSCLC. In addition, our results revealed that the combination of baseline CTCs and *hTERT* cfDNA levels is significantly associated with PFS and the response to pembrolizumab therapy in patients with metastatic NSCLC. Notably, using our approach, we were able to identify a subgroup of patients who were negative for CTCs, who presented low levels of *hTERT* cfDNA and who particularly benefited from the treatment. Early evaluation of the response to immunotherapy might enable clinicians to decide whether the clinical benefit is sufficient to continue treatment, avoiding unnecessary toxicities and costs.

## Conflict of interest

Jorge García‐González reports personal fees from AstraZeneca, Boehringer Ingelheim, Novartis, Pierre Fabre, Rovi and Sanofi; and personal fees and nonfinancial support from Bristol‐Myers Squibb, Lilly, MSD and Roche, outside the submitted work. Luis León‐Mateos reports personal fees from AstraZeneca, Boehringer Ingelheim, Novartis, Jansen, Astellas and Sanofi; and personal fees and nonfinancial support from Bristol‐Myers Squibb, Lilly, MSD and Roche, outside the submitted work. Santiago Aguín reports personal fees from Merck, MSD, Bristol‐Myers Squibb and Rovi; and personal fees and nonfinancial support from Kyowa Kirin outside the submitted work. Urbano Anido reports nonfinancial support and other from Pfizer; personal fees, nonfinancial support and other from Novartis; personal fees, nonfinancial support and other from Bayer; personal fees, nonfinancial support and other from Ipsen; other from EUSA; nonfinancial support and other from Sanofi; grants and nonfinancial support from Pierre Fabre; nonfinancial support and other from Advanced Accelerator Applications; personal fees and nonfinancial support from BMS; nonfinancial support from Roche; personal fees and nonfinancial support from Astellas; personal fees from Janssen; personal fees from Kyowa Kirin; and personal fees from Lilly, outside the submitted work. Rafael López‐López reports grants and personal fees from Roche, Merck, AstraZeneca, Bayer, Pharmamar and Leo; and personal fees and nonfinancial support from Bristol‐Myers Squibb and Novartis, outside of the submitted work.

## Author contributions

All authors were involved in drafting the article or revising it critically for important intellectual content, and all authors approved the final version. RD‐P takes responsibility for the integrity of the data and the accuracy of the data analysis. PM‐M contributed to conceptualization, methodology, investigation, data curation, software and analysis of data. JG‐G performed acquisition of data, investigation and data curation. LL‐M performed acquisition of data, investigation and data curation. UA and SA performed acquisition of data. IA, MS‐A and RML‐L contributed to methodology. AA and AR‐C contributed to methodology and data curation. AD‐L contributed to methodology and investigation. LM‐R and RL‐L contributed to conceptualization, project administration and funding acquisition. RD‐P contributed to study conception and design, analysis and interpretation of data, and supervision.

### Peer Review

The peer review history for this article is available at https://publons.com/publon/10.1002/1878‐0261.13094.

## Supporting information


**Fig. S1.** *hTERT* cfDNA changes during Pembrolizumab therapy and their association with disease progression. (A) cfDNA levels at different time‐points (baseline, 6 and 12 weeks); (B) cfDNA levels according the response to therapy. cfDNA, circulating‐free DNA.
**Fig. S2.** Immunofluorescence characterization of PD‐L1 in cancer cell lines.
**Fig. S3.** Concordance analysis between the detection of CTCs (A) and CTCs PD‐L1‐positive (B) using the CellSearch^®^ and Parsortix systems (Kappa test).
**Fig. S4.** Correlation of PD‐L1 positivity between tumor tissues (by tumor proportion scores) and CTCs with the CellSearch^®^ (A) and Parsortix systems (B).
**Fig. S5.** Objective response rate in patients with low cfDNA levels and undetectable CTCs (n = 12) versus patients with high cfDNA levels and undetectable CTCs or low cfDNA levels and detectable CTCs or high cfDNA levels and detectable CTCs (n = 18).
**Table S1.** ROC analysis to determine the value of *hTERT* cfDNA levels to discriminate progression or death.
**Table S2.** Circulating tumor cells enumeration and PD‐L1 analysed using CellSearch^®^ and Parsortix systems.
**Table S3.** Comparison of the CTCs levels according to the response to therapy.

## Data Availability

The data that support the findings of this study are available from the corresponding author (roberto.diaz.pena@sergas.es) upon reasonable request.

## References

[1] Siegel RL , Miller KD & Jemal A (2019) Cancer statistics, 2019. CA Cancer J Clin 69, 7–34. 30620402 10.3322/caac.21551

[2] Pardoll DM (2012) The blockade of immune checkpoints in cancer immunotherapy. Nat Rev Cancer 12, 252–264. 22437870 10.1038/nrc3239 PMC4856023

[3] Houston KA , Henley SJ , Li J , White MC & Richards TB (2014) Patterns in lung cancer incidence rates and trends by histologic type in the United States, 2004–2009. Lung Cancer 86, 22–28. 25172266 10.1016/j.lungcan.2014.08.001 PMC5823254

[4] Reck M , Rodríguez‐Abreu D , Robinson AG , Hui R , Csőszi T , Fülöp A , Gottfried M , Peled N , Tafreshi A , Cuffe S *et al*. (2016) Pembrolizumab versus chemotherapy for PD‐L1–positive non–small‐cell lung cancer. N Engl J Med 375, 1823–1833. 27718847 10.1056/NEJMoa1606774

[5] Planchard D , Popat S , Kerr K , Novello S , Smit EF , Faivre‐Finn C , Mok TS , Reck M , Van Schil PE , Hellmann MD *et al*. (2018) Metastatic non‐small cell lung cancer: ESMO Clinical Practice Guidelines for diagnosis, treatment and follow‐up. Ann Oncol 29, iv192–iv237. 10.1093/annonc/mdy275 30285222

[6] Blons H , Garinet S , Laurent‐Puig P & Oudart J‐B (2019) Molecular markers and prediction of response to immunotherapy in non‐small cell lung cancer, an update. J Thorac Dis 11, S25–S36. 30775025 10.21037/jtd.2018.12.48 PMC6353739

[7] Gandhi L , Rodríguez‐Abreu D , Gadgeel S , Esteban E , Felip E , De Angelis F , Domine M , Clingan P , Hochmair MJ , Powell SF *et al*. (2018) Pembrolizumab plus chemotherapy in metastatic non–small‐cell lung cancer. N Engl J Med 378, 2078–2092. 29658856 10.1056/NEJMoa1801005

[8] Paz‐Ares L , Luft A , Vicente D , Tafreshi A , Gümüş M , Mazières J , Hermes B , Çay Şenler F , Csőszi T , Fülöp A *et al*. (2018) Pembrolizumab plus chemotherapy for squamous non–small‐cell lung cancer. N Engl J Med 379, 2040–2051. 30280635 10.1056/NEJMoa1810865

[9] Rossi S , Castello A , Toschi L & Lopci E (2018) Immunotherapy in non‐small‐cell lung cancer: potential predictors of response and new strategies to assess activity. Immunotherapy 10, 797–805. 30008262 10.2217/imt-2017-0187

[10] Tellez‐Gabriel M , Heymann MF & Heymann D (2019) Circulating tumor cells as a tool for assessing tumor heterogeneity. Theranostics 9, 4580–4594. 31367241 10.7150/thno.34337 PMC6643448

[11] Russano M , Napolitano A , Ribelli G , Iuliani M , Simonetti S , Citarella F , Pantano F , Dell'Aquila E , Anesi C , Silvestris N *et al*. (2020) Liquid biopsy and tumor heterogeneity in metastatic solid tumors: the potentiality of blood samples. J Exp Clin Cancer Res 39, 120. 32580740 10.1186/s13046-020-01615-w PMC7313142

[12] Mondelo‐Macía P , Rodríguez‐López C , Valiña L , Aguín S , León‐Mateos L , García‐González J , Abalo A , Rapado‐González O , Suárez‐Cunqueiro M , Díaz‐Lagares A *et al*. (2020) Detection of MET alterations using cell free DNA and circulating tumor cells from cancer patients. Cells 9, 522. 10.3390/cells9020522 PMC7072825 32102486

[13] Brozos‐Vázquez EM , Díaz‐Peña R , García‐González J , León‐Mateos L , Mondelo‐Macía P , Peña‐Chilet M & López‐López R (2020) Immunotherapy in nonsmall‐cell lung cancer: current status and future prospects for liquid biopsy. Cancer Immunol Immunother 70, 1177–1188. 33113004 10.1007/s00262-020-02752-z PMC10991125

[14] Wit S , Rossi E , Weber S , Tamminga M , Manicone M , Swennenhuis JF , Groothuis‐Oudshoorn CGM , Vidotto R , Facchinetti A , Zeune LL *et al*. (2019) Single tube liquid biopsy for advanced non‐small cell lung cancer. Int J Cancer 144, 3127–3137. 30536653 10.1002/ijc.32056

[15] Alama A , Coco S , Genova C , Rossi G , Fontana V , Tagliamento M , Bello MGD , Rosa A , Boccardo S , Rijavec E *et al*. (2019) Prognostic relevance of circulating tumor cells and circulating cell‐free DNA association in metastatic non‐small cell lung cancer treated with nivolumab. J Clin Med 8, 1011. 10.3390/jcm8071011 PMC6679117 31295929

[16] Giroux Leprieur E , Herbretau G , Dumenil C , Julie C , Giraud V , Labrune S , Dumoulin J , Tisserand J , Emile J‐F , Blons H *et al*. (2018) Circulating tumor DNA evaluated by next‐generation sequencing is predictive of tumor response and prolonged clinical benefit with nivolumab in advanced non‐small cell lung cancer. Oncoimmunology 7, e1424675. 29721388 10.1080/2162402X.2018.1424675 PMC5927532

[17] Zhou X , Li C , Zhang Z , Li DY , Du J , Ding P , Meng H , Xu H , Li R , Ho E *et al*. (2021) Kinetics of plasma cfDNA predicts clinical response in non‐small cell lung cancer patients. Sci Rep 11, 7633. 33828112 10.1038/s41598-021-85797-z PMC8027214

[18] Sirera R , Bremnes RM , Cabrera A , Jantus‐Lewintre E , Sanmartín E , Blasco A , del Pozo N , Rosell R , Guijarro R , Galbis J *et al*. (2011) Circulating DNA is a useful prognostic factor in patients with advanced non‐small cell lung cancer. J Thorac Oncol 6, 286–290. 21252717 10.1097/JTO.0b013e31820189a5

[19] Mazel M , Jacot W , Pantel K , Bartkowiak K , Topart D , Cayrefourcq L , Rossille D , Maudelonde T , Fest T & Alix‐Panabières C (2015) Frequent expression of PD‐L1 on circulating breast cancer cells. Mol Oncol 9, 1773–1782. 26093818 10.1016/j.molonc.2015.05.009 PMC5528721

[20] Scheel AH , Baenfer G , Baretton G , Dietel M , Diezko R , Henkel T , Heukamp LC , Jasani B , Jöhrens K , Kirchner T *et al*. (2018) Interlaboratory concordance of PD‐L1 immunohistochemistry for non‐small‐cell lung cancer. Histopathology 72, 449–459. 28851100 10.1111/his.13375

[21] Robin X , Turck N , Hainard A , Tiberti N , Lisacek F , Sanchez J‐C & Müller M (2011) pROC: an open‐source package for R and S+ to analyze and compare ROC curves. BMC Bioinformatics 12, 77. 21414208 10.1186/1471-2105-12-77 PMC3068975

[22] Therneau TM & Grambsch PM (2000) Modeling Survival Data: Extending the Cox Model. Springer, New York, NY.

[23] Hellmann MD , Ciuleanu T‐E , Pluzanski A , Lee JS , Otterson GA , Audigier‐Valette C , Minenza E , Linardou H , Burgers S , Salman P *et al*. (2018) Nivolumab plus ipilimumab in lung cancer with a high tumor mutational burden. N Engl J Med 378, 2093–2104. 29658845 10.1056/NEJMoa1801946 PMC7193684

[24] Hellmann MD , Paz‐Ares L , Bernabe Caro R , Zurawski B , Kim S‐W , Carcereny Costa E , Park K , Alexandru A , Lupinacci L , de la Mora Jimenez E *et al*. (2019) Nivolumab plus ipilimumab in advanced non–small‐cell lung cancer. N Engl J Med 381, 2020–2031. 31562796 10.1056/NEJMoa1910231

[25] Gandara DR , Paul SM , Kowanetz M , Schleifman E , Zou W , Li Y , Rittmeyer A , Fehrenbacher L , Otto G , Malboeuf C *et al*. (2018) Blood‐based tumor mutational burden as a predictor of clinical benefit in non‐small‐cell lung cancer patients treated with atezolizumab. Nat Med 24, 1441–1448. 30082870 10.1038/s41591-018-0134-3

[26] Fenizia F , Pasquale R , Roma C , Bergantino F , Iannaccone A & Normanno N (2018) Measuring tumor mutation burden in non‐small cell lung cancer: tissue versus liquid biopsy. Transl Lung Cancer Res 7, 668–677. 30505711 10.21037/tlcr.2018.09.23 PMC6249618

[27] Chen YT , Seeruttun SR , Wu XY & Wang ZX (2019) Maximum somatic allele frequency in combination with blood‐based tumor mutational burden to predict the efficacy of atezolizumab in advanced non‐small cell lung cancer: a pooled analysis of the randomized POPLAR and OAK studies. Front Oncol 9, 1–9. 31921683 10.3389/fonc.2019.01432 PMC6929100

[28] Wood MA , Weeder BR , David JK , Nellore A & Thompson RF (2020) Burden of tumor mutations, neoepitopes, and other variants are weak predictors of cancer immunotherapy response and overall survival. Genome Med 12, 33. 32228719 10.1186/s13073-020-00729-2 PMC7106909

[29] Tissot C , Toffart A‐C , Villar S , Souquet P‐J , Merle P , Moro‐Sibilot D , Pérol M , Zavadil J , Brambilla C , Olivier M *et al*. (2015) Circulating free DNA concentration is an independent prognostic biomarker in lung cancer. Eur Respir J 46, 1773–1780. 26493785 10.1183/13993003.00676-2015

[30] Ai B , Liu H , Huang Y & Peng P (2016) Circulating cell‐free DNA as a prognostic and predictive biomarker in non‐small cell lung cancer. Oncotarget 7, 44583–44595. 27323821 10.18632/oncotarget.10069 PMC5190120

[31] Cargnin S , Canonico PL , Genazzani AA & Terrazzino S (2017) Quantitative analysis of circulating cell‐free DNA for correlation with lung cancer survival: a systematic review and meta‐analysis. J Thorac Oncol 12, 43–53. 27543256 10.1016/j.jtho.2016.08.002

[32] Trigg RM , Martinson LJ , Parpart‐Li S & Shaw JA (2018) Factors that influence quality and yield of circulating‐free DNA: a systematic review of the methodology literature. Heliyon 4, e00699. 30094369 10.1016/j.heliyon.2018.e00699 PMC6074610

[33] Paci M , Maramotti S , Bellesia E , Formisano D , Albertazzi L , Ricchetti T , Ferrari G , Annessi V , Lasagni D , Carbonelli C *et al*. (2009) Circulating plasma DNA as diagnostic biomarker in non‐small cell lung cancer. Lung Cancer 64, 92–97. 18804892 10.1016/j.lungcan.2008.07.012

[34] Pavan A , Boscolo Bragadin A , Calvetti L , Ferro A , Zulato E , Attili I , Nardo G , Dal Maso A , Frega S , Menin AG *et al*. (2021) Role of next generation sequencing‐based liquid biopsy in advanced non‐small cell lung cancer patients treated with immune checkpoint inhibitors: impact of STK11, KRAS and TP53 mutations and co‐mutations on outcome. Transl Lung Cancer Res 10, 202–220. 33569305 10.21037/tlcr-20-674 PMC7867770

[35] Guibert N , Jones G , Beeler JF , Plagnol V , Morris C , Mourlanette J , Delaunay M , Keller L , Rouquette I , Favre G *et al*. (2019) Targeted sequencing of plasma cell‐free DNA to predict response to PD1 inhibitors in advanced non‐small cell lung cancer. Lung Cancer 137, 1–6. 31518912 10.1016/j.lungcan.2019.09.005

[36] Khagi Y , Goodman AM , Daniels GA , Patel SP , Sacco AG , Randall JM , Bazhenova LA & Kurzrock R (2017) Hypermutated circulating tumor DNA: correlation with response to checkpoint inhibitor–based immunotherapy. Clin Cancer Res 23, 5729–5736. 28972084 10.1158/1078-0432.CCR-17-1439 PMC5678984

[37] Bratman SV , Yang SYC , Iafolla MAJ , Liu Z , Hansen AR , Bedard PL , Lheureux S , Spreafico A , Razak AA , Shchegrova S *et al*. (2020) Personalized circulating tumor DNA analysis as a predictive biomarker in solid tumor patients treated with pembrolizumab. Nat Cancer 1, 873–881. 10.1038/s43018-020-0096-5 35121950

[38] Ricciuti B , Jones G , Severgnini M , Alessi JV , Recondo G , Lawrence M , Forshew T , Lydon C , Nishino M , Cheng M *et al*. (2021) Early plasma circulating tumor DNA (ctDNA) changes predict response to first‐line pembrolizumab‐based therapy in non‐small cell lung cancer (NSCLC). J Immunother Cancer 9, e001504. 33771889 10.1136/jitc-2020-001504 PMC7996662

[39] Cabel L , Riva F , Servois V , Livartowski A , Daniel C , Rampanou A , Lantz O , Romano E , Milder M , Buecher B *et al*. (2017) Circulating tumor DNA changes for early monitoring of anti‐PD1 immunotherapy: a proof‐of‐concept study. Ann Oncol 28, 1996–2001. 28459943 10.1093/annonc/mdx212

[40] Goldberg SB , Narayan A , Kole AJ , Decker RH , Teysir J , Carriero NJ , Lee A , Nemati R , Nath SK , Mane SM *et al*. (2018) Early assessment of lung cancer immunotherapy response via circulating tumor DNA. Clin Cancer Res 24, 1872–1880. 29330207 10.1158/1078-0432.CCR-17-1341 PMC5899677

[41] Cheng C , Omura‐Minamisawa M , Kang Y , Hara T , Koike I & Inoue T (2009) Quantification of circulating cell‐free DNA in the plasma of cancer patients during radiation therapy. Cancer Sci 100, 303–309. 19200259 10.1111/j.1349-7006.2008.01021.x PMC11158820

[42] Gautschi O , Bigosch C , Huegli B , Jermann M , Marx A , Chassé E , Ratschiller D , Weder W , Joerger M , Betticher DC *et al*. (2004) Circulating deoxyribonucleic acid as prognostic marker in non‐small‐cell lung cancer patients undergoing chemotherapy. J Clin Oncol 22, 4157–4164. 15483026 10.1200/JCO.2004.11.123

[43] Sozzi G , Conte D , Leon ME , Cirincione R , Roz L , Ratcliffe C , Roz E , Cirenei N , Bellomi M , Pelosi G *et al*. (2003) Quantification of free circulating DNA as a diagnostic marker in lung cancer. J Clin Oncol 21, 3902–3908. 14507943 10.1200/JCO.2003.02.006

[44] Meddeb R , Dache ZAA , Thezenas S , Otandault A , Tanos R , Pastor B , Sanchez C , Azzi J , Tousch G , Azan S *et al*. (2019) Quantifying circulating cell‐free DNA in humans. Sci Rep 9, 1–16. 30914716 10.1038/s41598-019-41593-4 PMC6435718

[45] Brodbeck K , Schick S , Bayer B , Anslinger K , Krüger K , Mayer Z , Holdenrieder S & Peldschus S (2020) Biological variability of cell‐free DNA in healthy females at rest within a short time course. Int J Legal Med 134, 911–919. 31900625 10.1007/s00414-019-02240-9

[46] Cristofanilli M , Budd GT , Ellis MJ , Stopeck A , Matera J , Miller MC , Reuben JM , Doyle GV , Allard WJ , Terstappen LWMM *et al*. (2004) Circulating tumor cells, disease progression, and survival in metastatic breast cancer. N Engl J Med 351, 781–791. 15317891 10.1056/NEJMoa040766

[47] Cohen SJ , Punt CJA , Iannotti N , Saidman BH , Sabbath KD , Gabrail NY , Picus J , Morse M , Mitchell E , Miller MC *et al*. (2008) Relationship of circulating tumor cells to tumor response, progression‐free survival, and overall survival in patients with metastatic colorectal cancer. J Clin Oncol 26, 3213–3221. 18591556 10.1200/JCO.2007.15.8923

[48] de Bono JS , Scher HI , Montgomery RB , Parker C , Miller MC , Tissing H , Doyle GV , Terstappen LWWM , Pienta KJ & Raghavan D (2008) Circulating tumor cells predict survival benefit from treatment in metastatic castration‐resistant prostate cancer. Clin Cancer Res 14, 6302–6309. 18829513 10.1158/1078-0432.CCR-08-0872

[49] Yu M , Stott S , Toner M , Maheswaran S & Haber DA (2011) Circulating tumor cells: approaches to isolation and characterization. J Cell Biol 192, 373–382. 21300848 10.1083/jcb.201010021 PMC3101098

[50] Habli Z , AlChamaa W , Saab R , Kadara H & Khraiche ML (2020) Circulating tumor cell detection technologies and clinical utility: challenges and opportunities. Cancers (Basel) 12, 1–30. 10.3390/cancers12071930 PMC7409125 32708837

[51] Janning M , Kobus F , Babayan A , Wikman H , Velthaus J‐L , Bergmann S , Schatz S , Falk M , Berger L‐A , Böttcher L‐M *et al*. (2019) Determination of PD‐L1 expression in circulating tumor cells of NSCLC patients and correlation with response to PD‐1/PD‐L1 inhibitors. Cancers (Basel) 11, 835. 10.3390/cancers11060835 PMC6627043 31212989

[52] Kurokawa M , Ise N , Omi K , Goishi K & Higashiyama S (2013) Cisplatin influences acquisition of resistance to molecular‐targeted agents through epithelial‐mesenchymal transition‐like changes. Cancer Sci 104, 904–911. 23566288 10.1111/cas.12171 PMC7657214

[53] Jakobsen KR , Demuth C , Sorensen BS & Nielsen AL (2016) The role of epithelial to mesenchymal transition in resistance to epidermal growth factor receptor tyrosine kinase inhibitors in non‐small cell lung cancer. Transl Lung Cancer Res 5, 172–182. 27186512 10.21037/tlcr.2016.04.07 PMC4858579

[54] Fukuda K , Takeuchi S , Arai S , Katayama R , Nanjo S , Tanimoto A , Nishiyama A , Nakagawa T , Taniguchi H , Suzuki T *et al*. (2019) Epithelial‐to‐mesenchymal transition is a mechanism of ALK inhibitor resistance in lung cancer independent of ALK mutation status. Cancer Res 79, 1658–1670. 30737231 10.1158/0008-5472.CAN-18-2052

[55] Zhu X , Chen L , Liu L & Niu X (2019) EMT‐mediated acquired EGFR‐TKI resistance in NSCLC: mechanisms and strategies. Front Oncol 9, 1044. 31681582 10.3389/fonc.2019.01044 PMC6798878

[56] Chudziak J , Burt DJ , Mohan S , Rothwell DG , Mesquita B , Antonello J , Dalby S , Ayub M , Priest L , Carter L *et al*. (2016) Clinical evaluation of a novel microfluidic device for epitope‐independent enrichment of circulating tumour cells in patients with small cell lung cancer. Analyst 141, 669–678. 26605519 10.1039/c5an02156a

[57] Ilié M , Szafer‐Glusman E , Hofman V , Chamorey E , Lalvée S , Selva E , Leroy S , Marquette C‐H , Kowanetz M , Hedge P *et al*. (2018) Detection of PD‐L1 in circulating tumor cells and white blood cells from patients with advanced non‐small‐cell lung cancer. Ann Oncol 29, 193–199. 29361135 10.1093/annonc/mdx636

[58] Guibert N , Delaunay M , Lusque A , Boubekeur N , Rouquette I , Clermont E , Mourlanette J , Gouin S , Dormoy I , Favre G *et al*. (2018) PD‐L1 expression in circulating tumor cells of advanced non‐small cell lung cancer patients treated with nivolumab. Lung Cancer 120, 108–112. 29748004 10.1016/j.lungcan.2018.04.001

[59] Boffa DJ , Graf RP , Salazar MC , Hoag J , Lu D , Krupa R , Louw J , Dugan L , Wang Y , Landers M *et al*. (2017) Cellular expression of PD‐L1 in the peripheral blood of lung cancer patients is associated with worse survival. Cancer Epidemiol Biomarkers Prev 26, 1139–1145. 28446544 10.1158/1055-9965.EPI-17-0120 PMC5500408

[60] Nicolazzo C , Raimondi C , Mancini M , Caponnetto S , Gradilone A , Gandini O , Mastromartino M , del Bene G , Prete A , Longo F *et al*. (2016) Monitoring PD‐L1 positive circulating tumor cells in non‐small cell lung cancer patients treated with the PD‐1 inhibitor Nivolumab. Sci Rep 6, 31726. 27553175 10.1038/srep31726 PMC4995431

[61] Dhar M , Wong J , Che J , Matsumoto M , Grogan T , Elashoff D , Garon EB , Goldman JW , Sollier Christen E , Di Carlo D *et al*. (2018) Evaluation of PD‐L1 expression on vortex‐isolated circulating tumor cells in metastatic lung cancer. Sci Rep 8, 2592. 29416054 10.1038/s41598-018-19245-w PMC5803213

[62] Cheng Y , Wang T , Lv X , Li R , Yuan L , Shen J , Li Y , Yan T , Liu B & Wang L (2020) Detection of PD‐L1 expression and its clinical significance in circulating tumor cells from patients with non‐small‐cell lung cancer. Cancer Manag Res 12, 2069–2078. 32256114 10.2147/CMAR.S245425 PMC7093656

[63] Koh Y , Yagi S , Akamatsu H , Kanai K , Hayata A , Tokudome N , Akamatsu K , Higuchi M , Kanbara H , Nakanishi M *et al*. (2019) Heterogeneous expression of programmed death receptor‐ligand 1 on circulating tumor cells in patients with lung cancer. Clin Lung Cancer 20, 270–277. 31005568 10.1016/j.cllc.2019.03.004

[64] Guibert N , Pradines A , Mazieres J & Favre G (2020) Current and future applications of liquid biopsy in nonsmall cell lung cancer from early to advanced stages. Eur Respir Rev 29, 190052. 32051167 10.1183/16000617.0052-2019 PMC9488537

[65] Jacot W , Mazel M , Mollevi C , Pouderoux S , D’Hondt V , Cayrefourcq L , Bourgier C , Boissiere‐Michot F , Berrabah F , Lopez‐Crapez E *et al*. (2020) Clinical correlations of programmed cell death ligand 1 status in liquid and standard biopsies in breast cancer. Clin Chem 66, 1093–1101. 32712650 10.1093/clinchem/hvaa121

[66] Haragan A , Field JK , Davies MPA , Escriu C , Gruver A & Gosney JR (2019) Heterogeneity of PD‐L1 expression in non‐small cell lung cancer: implications for specimen sampling in predicting treatment response. Lung Cancer 134, 79–84. 31320000 10.1016/j.lungcan.2019.06.005 PMC6658831

[67] Lindsay CR , Blackhall FH , Carmel A , Fernandez‐Gutierrez F , Gazzaniga P , Groen H , Hiltermann T , Krebs MG , Loges S , López‐López R *et al*. (2019) EPAC‐lung: pooled analysis of circulating tumour cells in advanced non‐small cell lung cancer. Eur J Cancer 117, 60–68. 31254940 10.1016/j.ejca.2019.04.019

[68] Alidousty C , Duerbaum N , Wagener‐Ryczek S , Baar T , Martelotto LG , Heydt C , Siemanowski J , Holz B , Binot E , Fassunke J *et al*. (2021) Prevalence and potential biological role of TERT amplifications in ALK translocated adenocarcinoma of the lung. Histopathology 78, 578–585. 32946634 10.1111/his.14256

[69] Barthel FP , Wei W , Tang M , Martinez‐Ledesma E , Hu X , Amin SB , Akdemir KC , Seth S , Song X , Wang Q *et al*. (2017) Systematic analysis of telomere length and somatic alterations in 31 cancer types. Nat Genet 49, 349–357. 28135248 10.1038/ng.3781 PMC5571729

