# Supplementary material for: Clinical potential of circulating free DNA and circulating tumour cells in patients with metastatic non‐small‐cell lung cancer treated with pembrolizumab
Source: Mol Oncol. 2021 Sep 23;15(11):2923–40. doi: 10.1002/1878-0261.13094 (PMC8564635; doi:10.1002/1878-0261.13094)
Supplement: Supplementary file 1 — Fig. S1. hTERT cfDNA changes during Pembrolizumab therapy and their association with disease progression. (A) cfDNA levels at different time‐points (baseline, 6 and 12 weeks); (B) cfDNA levels according the response to therapy. cfDNA, circulating‐free DNA. Fig. S2. Immunofluorescence characterization of PD‐L1 in cancer cell lines. Fig. S3. Concordance analysis between the detection of CTCs (A) and CTCs PD‐L1‐positive (B) using the CellSearch® and Parsortix systems (Kappa test). Fig. S4. Correlation of PD‐L1 positivity between tumor tissues (by tumor proportion scores) and CTCs with the CellSearch® (A) and Parsortix systems (B). Fig. S5. Objective response rate in patients with low cfDNA levels and undetectable CTCs (n = 12) versus patients with high cfDNA levels and undetectable CTCs or low cfDNA levels and detectable CTCs or high cfDNA levels and detectable CTCs (n = 18). Table S1. ROC analysis to determine the value of hTERT cfDNA levels to discriminate progression or death. Table S2. Circulating tumor cells enumeration and PD‐L1 analysed using CellSearch® and Parsortix systems. Table S3. Comparison of the CTCs levels according to the response to therapy. [file MOL2-15-2923-s001.doc]

**Table S1**. ROC analysis to determine the value of *hTERT* cfDNA levels to discriminate progression or death.

| **Parameters** | **AUROC** | **Threshold** | **Sensitivity** | **Specificity** |
| --- | --- | --- | --- | --- |
| ***PFS*** | | | | |
| Log cfDNA at baseline | 0.535 | 7.665 | 0.538 | 0.667 |
| Log cfDNA at 6 weeks | 0.600 | 7.336 | 0.722 | 0.533 |
| Log cfDNA at 12 weeks | 0.810 | 7.026 | 0.857 | 0.750 |
| ***OS*** | | | | |
| Log cfDNA at baseline | 0.650 | 7.638 | 0.640 | 0.720 |
| Log cfDNA at 6 weeks | 0.617 | 6.716 | 0.929 | 0.368 |
| Log cfDNA at 12 weeks | 0.746 | 7.026 | 0.818 | 0.600 |

Abbreviations: cfDNA, circulating-free DNA; PFS, progression-free survival; OS, overall survival; ROC, receiver operating characteristic; AUROC, area under the ROC curve.

**Figure S1.** *hTERT* cfDNA changes during Pembrolizumab therapy and their association with disease progression. (A) cfDNA levels at different time-points (baseline, 6 and 12 weeks); (B) cfDNA levels according the response to therapy. cfDNA, circulating-free DNA.


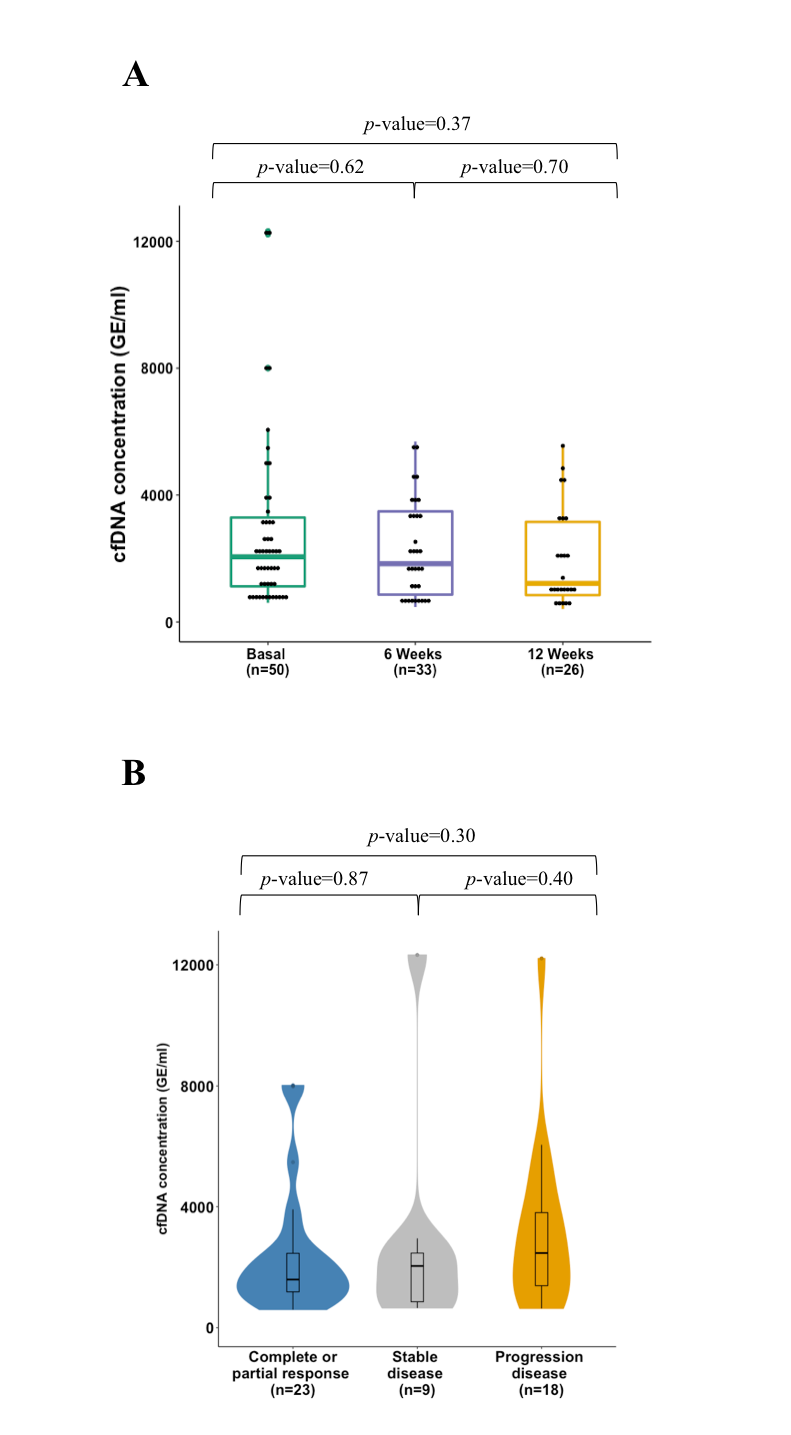


**Figure S2.** Immunofluorescence characterization of PD-L1 in cancer cell lines. We used three lung cancer cell lines with different grade of PD-L1 expression (NCI-H460, medium-high expression, NCI-H322, low-medium expression and A549, no expression).


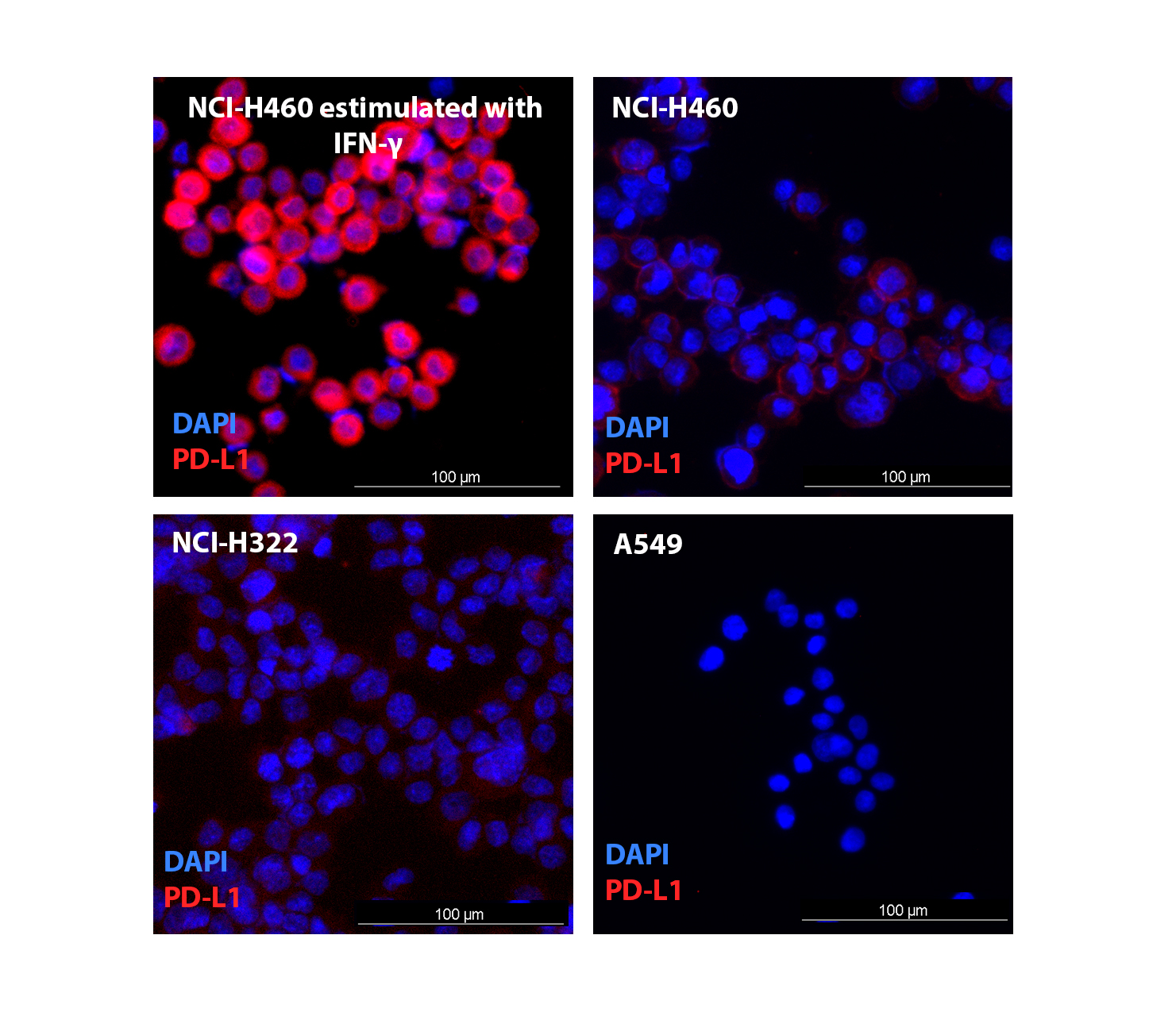


**Figure S3.** Concordance analysis between the detection of CTCs (A) and CTCs PD-L1-positive (B) using the CellSearch® and Parsortix systems (Kappa test).


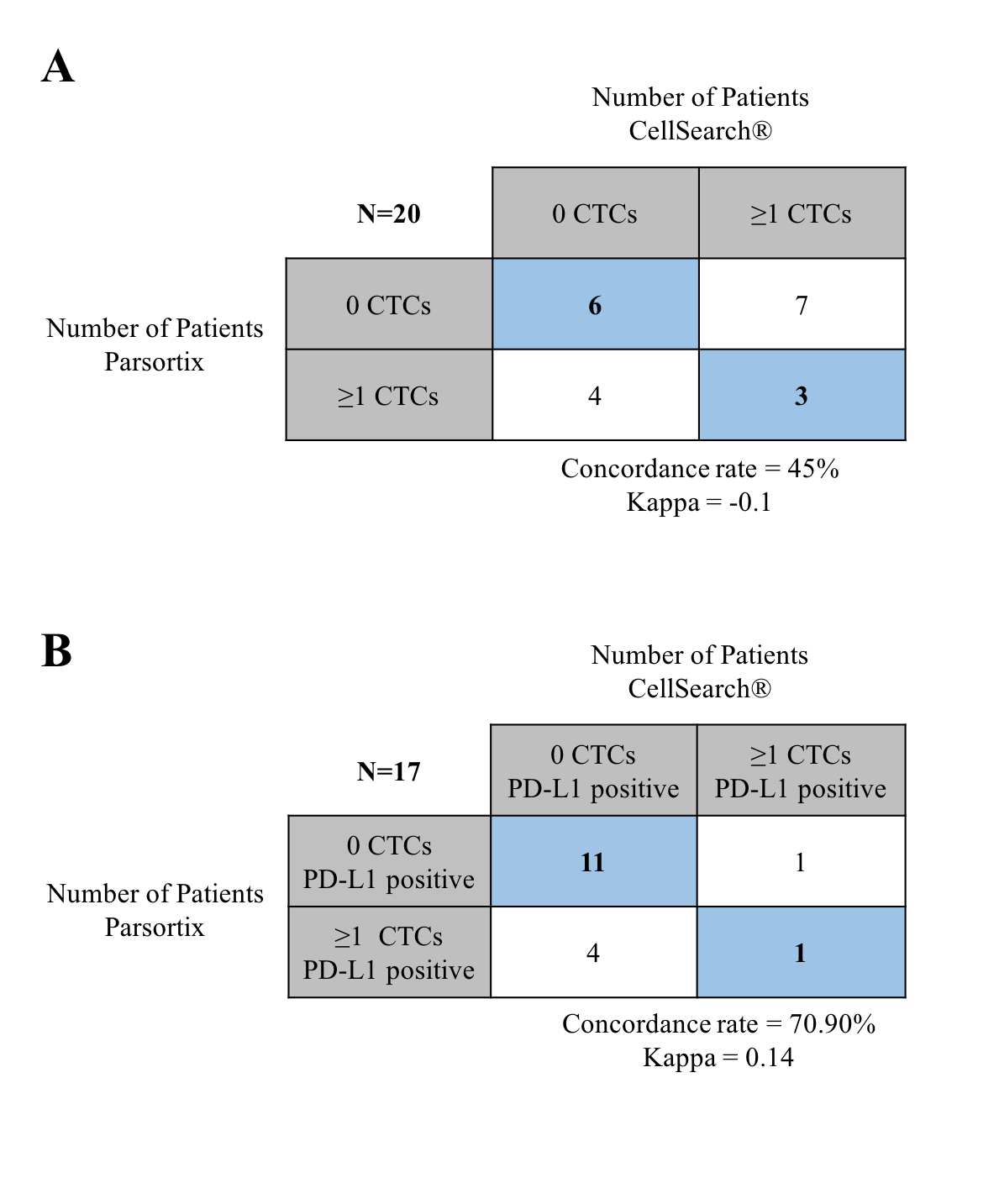


**Table S2.** Circulating tumor cells enumeration and PD-L1 analyzed using CellSearch® and Parsortix systems.

| **Sample ID** | **Parsortix System** | |  |  | **CellSearch® System** | | |  |  |
| --- | --- | --- | --- | --- | --- | --- | --- | --- | --- |
|  | **CTCs**  ***Total* *number**** | **PD-L1^+^ CTCs *Total Number* (%)*** |  |  | | **CTCs**  ***Total* *number**** | **PD-L1^+^ CTCs *Total Number* (%)*** |  |  |
|  |  |  |  |  |  |  |  |  |  |
| id1 | 9 | 5 (55.55) |  |  | | 0 | 0 (0.00) |  |  |
| id2 | 13 | 9 (69.23) |  |  | | 0 | 0 (0.00) |  |  |
| id3 | 8 | 6 (75.00) |  |  | | 0 | 0 (0.00) |  |  |
| id4 | 2 | 1 (50.00) |  |  | | 5 | NT |  |  |
| id5 | 5 | 5 (100.00) |  |  | | 3 | NT |  |  |
| id6 | 0 | 0 (0.00) |  |  | | 2 | NT |  |  |
| id7 | 0 | 0 (0.00) |  |  | | 0 | 0 (0.00) |  |  |
| id8 | 0 | 0 (0.00) |  |  | | 4 | 0 (0.00) |  |  |
| id9 | 0 | 0 (0.00) |  |  | | 2 | 0 (0.00) |  |  |
| id10 | 0 | 0 (0.00) |  |  | | 0 | 0 (0.00) |  |  |
| id11 | 56 | 18 (32.14) |  |  | | 168 | 7 (4.16) |  |  |
| id12 | 0 | 0 (0.00) |  |  | | 1 | 0 (0.00) |  |  |
| id13 | 0 | 0 (0.00) |  |  | | 6 | 0 (0.00) |  |  |
| id14 | 0 | 0 (0.00) |  |  | | 1 | 1 (100.00) |  |  |
| id15 | 0 | 0 (0.00) |  |  | | 0 | 0 (00.00) |  |  |
| id16 | 1 | 1 (100.00) |  |  | | 0 | 0 (00.00) |  |  |
| id17 | 0 | 0 (0.00) |  |  | | 0 | 0 (0.00) |  |  |
| id18 | 0 | 0 (0.00) |  |  | | 3 | 0 (0.00) |  |  |
| id19 | 0 | 0 (0.00) |  |  | | 0 | 0 (0.00) |  |  |
| id20 | 0 | 0 (0.00) |  |  | | 0 | 0 (0.00) |  |  |
| id21 | NT | NT |  |  | | 0 | 0 (0.00) |  |  |
| id22 | NT | NT |  |  | | 0 | 0 (0.00) |  |  |
| id23 | NT | NT |  |  | | 0 | 0 (0.00) |  |  |
| id24 | NT | NT |  |  | | 0 | 0 (0.00) |  |  |
| id25 | NT | NT |  |  | | 1 | NT |  |  |
| id26 | NT | NT |  |  | | 0 | 0 (0.00) |  |  |
| id27 | NT | NT |  |  | | 0 | 0 (0.00) |  |  |
| id28 | NT | NT |  |  | | 0 | 0 (0.00) |  |  |
| id29 | NT | NT |  |  | | 0 | 0 (0.00) |  |  |
| id30 | NT | NT |  |  | | 0 | 0 (0.00) |  |  |

Abbreviations: NT, not tested. *Total CTCs count in 7.5mL of peripheral blood.

**Figure S4.** Correlation of PD-L1 positivity between tumor tissues (by tumor proportion scores) and CTCs with the CellSearch® (A) and Parsortix systems (B).


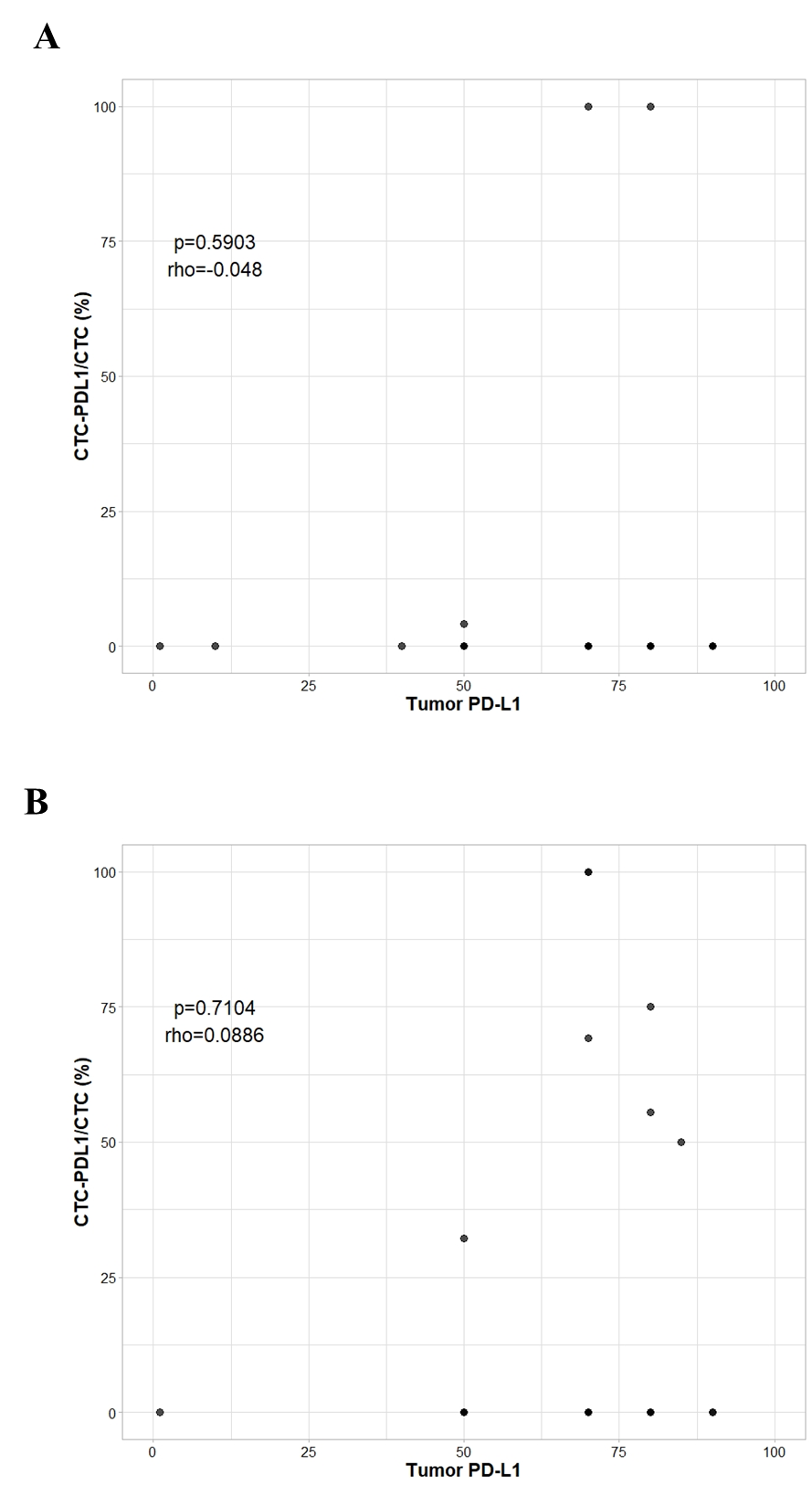


**Table S3.** Comparison of the CTCs levels according to the response to therapy.

| **CTCs** | **Complete**  **response**  ***n (%)*** | **Partial**  **response**  ***n (%)*** | **Stable**  **disease**  ***n (%)*** | **Progressive**  **disease**  ***n (%)*** | **Objective**  **response ratio**  ***n (%)*** |
| --- | --- | --- | --- | --- | --- |
| ***CellSearch® n=30*** |  |  |  |  |  |
| Undetectable CTCs, n=19 | 1 (5.26) | 10 (52.63) | 3 (16.67) | 5 (15.79) | 11 (57.89) |
| Detectable CTCs n=11 | 0 (0.00) | 2 (18.18) | 1 (9.09) | 8 (72.72) | 2 (18.18) |
| *CTCs-PDL1-negative, n=6** | 0 (0.00) | 1 (16.66) | 0 (0.00) | 5 (83.33) | 1 (16.66) |
| *CTCs-PDL1-positive, n=2** | 0 (0.00) | 1 (50.00) | 0 (0.00) | 1 (50.00) | 1 (50.00) |
| ***Parsortix n=20*** |  |  |  |  |  |
| Undetectable CTCs, n=13 | 0 (0.00) | 4 (30.77) | 2 (15.39) | 7 (53.86) | 4 (30.77) |
| Detectable CTCs n=7 | 0 (0.00) | 2 (28.57) | 1 (14.29) | 4 (57.14) | 2 (28.57) |
| *CTCs-PDL1-negative, n=5* | 0 (0.00) | 2 (40.00) | 1 (20.00) | 2 (40.00) | 2 (40.00) |
| *CTCs-PDL1-positive, n=7* | 0 (0.00) | 2 (28.57) | 1 (14.29) | 4 (57.14) | 2 (28.57) |

Objective response ratio: complete response and partial response during ≥6 cycles. *The detection of PD-L1 expression on CTCs using CellSearch® system was unrealizable in 4 patients.

**Figure S5.** Objective response rate in patients with low cfDNA levels and undetectable CTCs (n=12) versus patients with high cfDNA levels and undetectable CTCs or low cfDNA levels and detectable CTCs or high cfDNA levels and detectable CTCs (n=18).


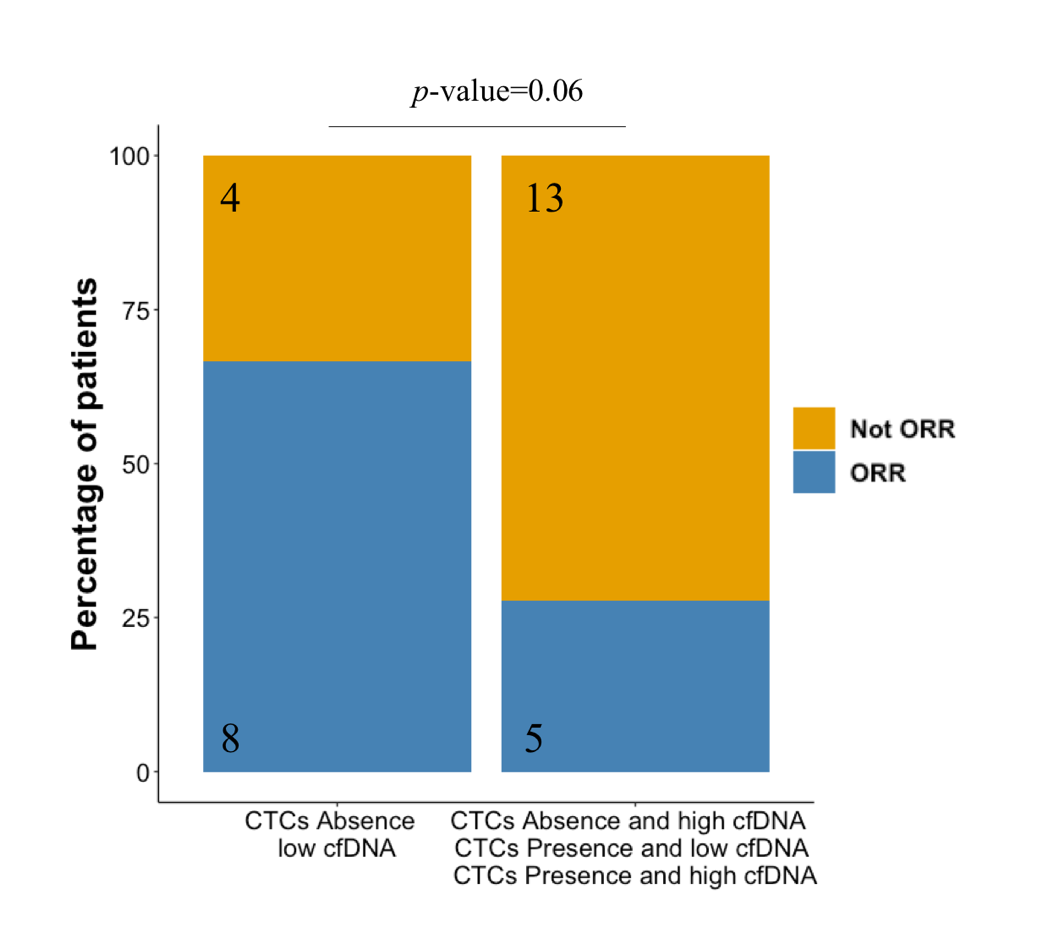


Abbreviations: cfDNA, circulating-free DNA; CTCs, circulating tumor cells; ORR, objective response ratio.
